# Oceanic islands act as drivers for the genetic diversity of marine species: *Cardita calyculata* (Linnaeus, 1758) in the NE Atlantic as a case-study

**DOI:** 10.1186/s12862-024-02322-2

**Published:** 2024-11-07

**Authors:** Livia Sinigaglia, L Baptista, C Alves, F Feldmann, C Sacchetti, C Rupprecht, T Vijayan, E Martín-González, SP Ávila, AM Santos, M Curto, H Meimberg

**Affiliations:** 1 Institute of Integrative Nature Conservation Research, Department of Integrative Biology and Biodiversity Research, University of Natural Resources and Life Sciences (BOKU), Vienna, Austria; 2 CIBIO, Centro de Investigação em Biodiversidade e Recursos Genéticos, InBIO Laboratório Associado, Pólo dos Açores, Ponta Delgada, Azores, 9501-801 Portugal; 3 MPB-Marine Palaeontology and Biogeography Lab, University of the Azores, Rua da Mãe de Deus, Ponta Delgada, Azores, 9501-801 Portugal; 4 Faculdade de Ciências da Universidade do Porto, Rua do Campo Alegre 1021/1055, Porto, 4169-007 Portugal; 5 Departamento de Biologia, Faculdade de Ciências e Tecnologia, Universidade dos Açores, Ponta Delgada, Azores, 9501-801 Portugal; 6 CIBIO, Centro de Investigação em Biodiversidade e Recursos Genéticos, InBIO Laboratório Associado, Universidade do Porto, Campus de Vairão, Rua Padre Armando Quintas, no. 7, Vairão, 4485-661 Portugal; 7 NIOZ Royal Netherlands Institute for Sea Research, Landsdiep 4 1797 SZ ’t Horntje, Texel, Netherlands; 8 Museo de Ciencias Naturales de Tenerife, Organismo Autónomo de Museos y Centros, C/ Fuente Morales, 1, 38003, Santa Cruz de Tenerife, Canary Islands Spain

**Keywords:** Marine island biogeography, Population genetic structure, Marine bivalve, Oceanic archipelagos

## Abstract

Geographic distribution, as well as evolutionary and biogeographic processes and patterns of marine invertebrate benthic species are strongly shaped by dispersal ability during the life cycle. Remote oceanic islands lie at the brink of complex biotic and abiotic interactions which have significantly influenced the biodiversity patterns we see today. The interaction between geological environmental change and taxon-specific dispersal modes can influence species evolutionary patterns, eventually delimiting species-specific biogeographic regions. In this study, we compare the population genetic patterns of the marine bivalve *Cardita calyculata* in the northeast Atlantic, discussing the role of Macaronesian islands during past climatic cycles. The genetic structure and diversity patterns were outlined based on SSR-GBAS loci of 165 individuals and on the mitochondrial COI marker of 22 individuals from the Canary Islands, Madeira, Azores and the Mediterranean. The highly structured genetic pattern found among regions and within archipelagos suggests the central role of oceanic islands in promoting the divergence of the species in both the NE Atlantic and the Mediterranean. The high degree of divergence in the COI dataset (> 7%) suggests the existence of potential cryptic speciation that needs to be further explored with a more comprehensive sampling. Such patterns are only congruent with a scenario where *C. calyculata* populations were maintained during glacial/interglacial cycles, supporting the role of the studied archipelagos as drivers of diversity for marine biota. We stress the importance of developing studies for species with various life history and dispersal modes. In such a way, a more profound understanding of the biogeographic and evolutionary significance of oceanic islands can catalyse directed conservation efforts, especially in the context of the ongoing climate crisis.

**Supplementary Information:**

The online version contains supplementary material available at 10.1186/s12862-024-02322-2.

## Background

The understanding of marine system dynamics greatly depends on knowing how climate and biotic factors shape biodiversity and evolutionary divergence at various geographical scales. This knowledge allows the inference of biogeographical processes and patterns for marine populations, which are essential to define biogeographic regions and respective boundaries.

For marine benthic invertebrates, geographic distribution, evolution and biogeography is strongly shaped by dispersal ability during the life cycle [1, 2]. Still, the complexity of biotic and abiotic factors shaping genetic exchanges in the marine realm and limited knowledge about larval development of most marine invertebrate species hamper a deep understanding of dispersal pathways and processes of marine benthic invertebrates, especially in remote islands. The dispersal capacity of marine invertebrates in early or late-life stages highly influences their distribution and gene flow. Species with planktotrophic larvae (i.e., with a long free-swimming feeding stage) can easily expand their distribution ranges and maintain gene flow by natural means during the larvae’s pelagic life [3, 4], which contributes to a genetic homogenization across wide geographical ranges [5–7]. On the other hand, restricted ranges are expected for species with non-planktotrophic larvae (i.e., direct development or production of lecithotrophic larvae that depend on yolk reserves for nutrition and energy until settlement) in consequence of a short period in the water column [1–4, 8–10]. Later on, dispersal of juveniles or adults is possible by several mechanisms, depending on their life-history traits. For species with low motility, passive dispersal is the most common way of exchange between distant populations (see [11] and references therein for a review). Several biotic and abiotic factors affect the likelihood of dispersal, thus shaping gene flow and patterns of genetic structure in benthic marine invertebrates with low motility [1, 12–14]. Regarding habitat type, intertidal species or those associated to algal patches are more likely to engage in passive dispersal following disturbance by natural mechanical action (e.g., waves), when compared to species in subtidal/circalittoral habitats or those sheltered in bare rocky substrata or sandy patches [1, 12, 15]. Sea-surface circulation is another factor influencing the direction and success of marine benthic invertebrates’ exchanges, by creating permanent or transient pathways/barriers for dispersal [16–18].

Contextualized to global timescales, glacial/interglacial cycles can have a deep influence on ocean dynamics and consequent geographic distribution of biodiversity [19]. Although global glaciation events have been common throughout the Earth’s history, they have increased in both duration and intensity since the beginning of the Pleistocene, around 2.58 Ma (millions of years ago) [1]. During the last few million years, the growth and retreat of ice sheets and glaciers, consequent to alternating cold stadial and warm interstadial events [20], triggered eustatic sea-level oscillations that exceeded amplitudes of 120–130 m [21]. Such events were most abrupt and pronounced across much of the Northern Hemisphere [20] and contributed to significantly change the North Atlantic thermohaline circulation [22]. The shift between glacial and interglacial conditions is marked by short-term events designated as Terminations; these events have a strong impact on regular sea-surface currents, changing or even, in some cases, reversing their usual course [23]. Temporary “windows of opportunity” are suggested by several authors to have been created during the final phase of glacial Terminations, allowing the geographical expansion/long-distance exchanges of marine biota [19, 21, 23–26], and potentially representing essential occasions for the survival and diversification of a species through geological time. Therefore, eustatic variations during the Pleistocene, had a profound effect on the dispersal and connectivity of marine populations, having shaped their current distribution patterns [19].

Some biodiversity-rich spots experienced minimal change during glacial periods providing a safe harbour for species over geological times and serving as a source for colonizers when the climate warmed [27]. Understanding the influence of these spots on current marine biodiversity patterns is thus essential for implementing efficient long-term conservation strategies as they may continue to provide refugia under anthropogenic climate change. Even if these high-biodiversity sites are not excluded from the impact of climate changes, they may slow down species extinction and turnover rates, and increase the chances for species to adapt and thus, enable their conservation [28]. Although terrestrial species are relatively well studied in oceanic islands, the same cannot be said for the marine realm. Understanding how specific dispersal modes interact with ecological habitat variation through geological time to shape species population structure, might thus shed light on the evolutionary processes behind long-term adaptations and survival of populations in the face of climate change. Oceanic islands represent a valuable natural laboratory for contextualizing such investigations. Characterized by rapid and intense ecological, climatic and evolutionary changes from continuous volcanic and erosional processes [19, 29], these discrete geographical entities, have prompted the inference of some of the most relevant evolutionary theories of the 19th century, starting with the fundamental contributions of Charles Darwin, Joseph Dalton Hooker and Alfred Russel Wallace [30–32]. Pleistocene climatic variations have played essential roles in altering littoral area (sensu [19]), elevation and effective degree of isolation of oceanic islands [33–35], often having significantly different ecological and evolutionary impacts than in nearby continental areas [21]. In particular, eustatic oscillations induced significant variations in habitat availability, by submerging islands or elevating seamounts and creating potential ‘stepping-stone’ routes of dispersal within the ocean [36].

The complex biogeographic processes in oceanic islands are reflected in the five oceanic volcanic archipelagos from the NE Atlantic - Azores, Madeira, Selvagens, Canaries, and Cabo Verde, also known as Macaronesia. This geographic area is renowned for its biodiversity in both the terrestrial and marine realms, which has prompted local governments to set marine and terrestrial conservation priorities [37]. Moreover, the geological dynamic of these archipelagos, the varying degree of isolation, latitudinal gradient and correlated climatic variation, together with the fact that these islands have never been connected with the mainland, make the NE Atlantic Archipelagos an ideal region to test biogeographic and evolutionary theories [38]. Large geographical distance, high differences in mean surface temperatures (SSTs, between 17 °C north of the Azores to 24 °C south of Cabo Verde), and oceanographic circulation in the NE Atlantic are expected to pose barriers to long-distance exchanges among NE Atlantic archipelagos. As a result, the Azores and Webbnesia (Madeira, Selvagens, and Canaries) constitute distinct ecoregions of the Lusitanian Province, whereas the Cabo Verdean islands are a biogeographic subprovince within the separate West African Transition Province [38]. Nevertheless, long-distance exchanges can happen with occasional dispersal events during extreme weather conditions [11–13, 39]. Several authors have also argued in favour of “windows of opportunity” associated with glacial Terminations during geological times that could have increased the exchange [19, 23–26, 40, 41]. Termination II (132–126 ky) and Termination I (19,5–6 ky) are, for example, considered as to have significantly influenced the biodiversity and biogeographic patterns we see today in the north-east Atlantic. In both cases, sea level rose by 120–130 m. Temporary sea-surface currents consequent to these events, favoured the range expansion of many marine species and the arrival of species to the Azores, mainly from Cabo Verde and the Canary Islands, but also from the east Atlantic [24–26].

*Cardita calyculata* (Linnaeus, 1758) is an epibenthic marine bivalve belonging to Carditida, an order of exclusively marine and predominantly suspension-feeders [42]. Like other carditid members, *C. calyculata* is epibyssate and attaches to intertidal and subtidal rocky-shore structures via the formation of byssus [43]. Most members of the family Carditidae are brooders [44–48] and juveniles are retained within the body cavity of the female until shell secretion commences and the prodissoconch has completely formed [49]. Although no direct observation of *C. calyculata* ontological development exists to date, the species exhibits features, such as a very invaginated byssal gape and sexual dimorphism, that have been shown to correlate to internal brooding [43]. In the NE Atlantic, *C. calyculata* has been reported from the Azores, Madeira, Selvagens, the Canaries and Cabo Verde archipelagos, Galicia, Portuguese coasts, Atlantic Moroccan shores, and various locations in the Mediterranean [50].

Intraspecific analyses of genetic differentiation and gene flow are significantly improved by high throughput sequencing methods, making them an effective tool for detecting the effects of geological and environmental factors on species dispersal, population connectivity and archipelago colonization processes. One of these methods allows the use of the whole sequence information of a genotyping marker and had been recently introduced as SSR-GBAS (SSR - Genotyping by Amplicon Sequencing) approach [51, 52]. By considering Whole Amplicon Information (WAI) genotypic data, this approach systematically surveys the entire sequence, summarizing the variability from both the repetition motif and single nucleotide polymorphisms (SNPs) in the flanking regions [52]. Consequently, increased information content can be viewed compared to traditional methods, enabling the determination of genetic structure in population genetic studies [13].

In the present study we genotyped 21 newly developed SSR-GBAS markers for 165 *C. calyculata* individuals from various locations in the Azores, Madeira, the Canaries and the Mediterranean. The analysis was complemented by checking the variation of mitochondrial DNA at the cytochrome c oxidase subunit 1 (COI) region to determine haplotype differentiation between populations. With this study, we aim to understand how the genetic population structure of a marine invertebrate with limited dispersal ability reflects the impact of glacial/interglacial cycles and of diversification between oceanic islands and mainland populations. The imprint of past geological and climate changes in its genetic and biogeographic patterns will be inferred by comparing two differently evolving regions in the genome. We expect that the limited dispersal potential represented by a brooder with direct development may interact with environmental oceanographic variations through geological time and climatic shifts to determine specific population genetic structures throughout the studied area. As such investigation is being contextualized within the ecologically and geologically dynamic environment of oceanic islands, specific population structures may arise which may help elucidate the role of the NE Atlantic archipelagos in both preserving and promoting the diversity of *C. calyculata* through time. The use of SSR-GBAS methods is herein of great value as this method enables the detection of genetic structures at small geographical scales allowing inferences on the influence of habitat and climatology on the retrieved patterns.

## Methodology

### Sample collection

**Fig. 1:**
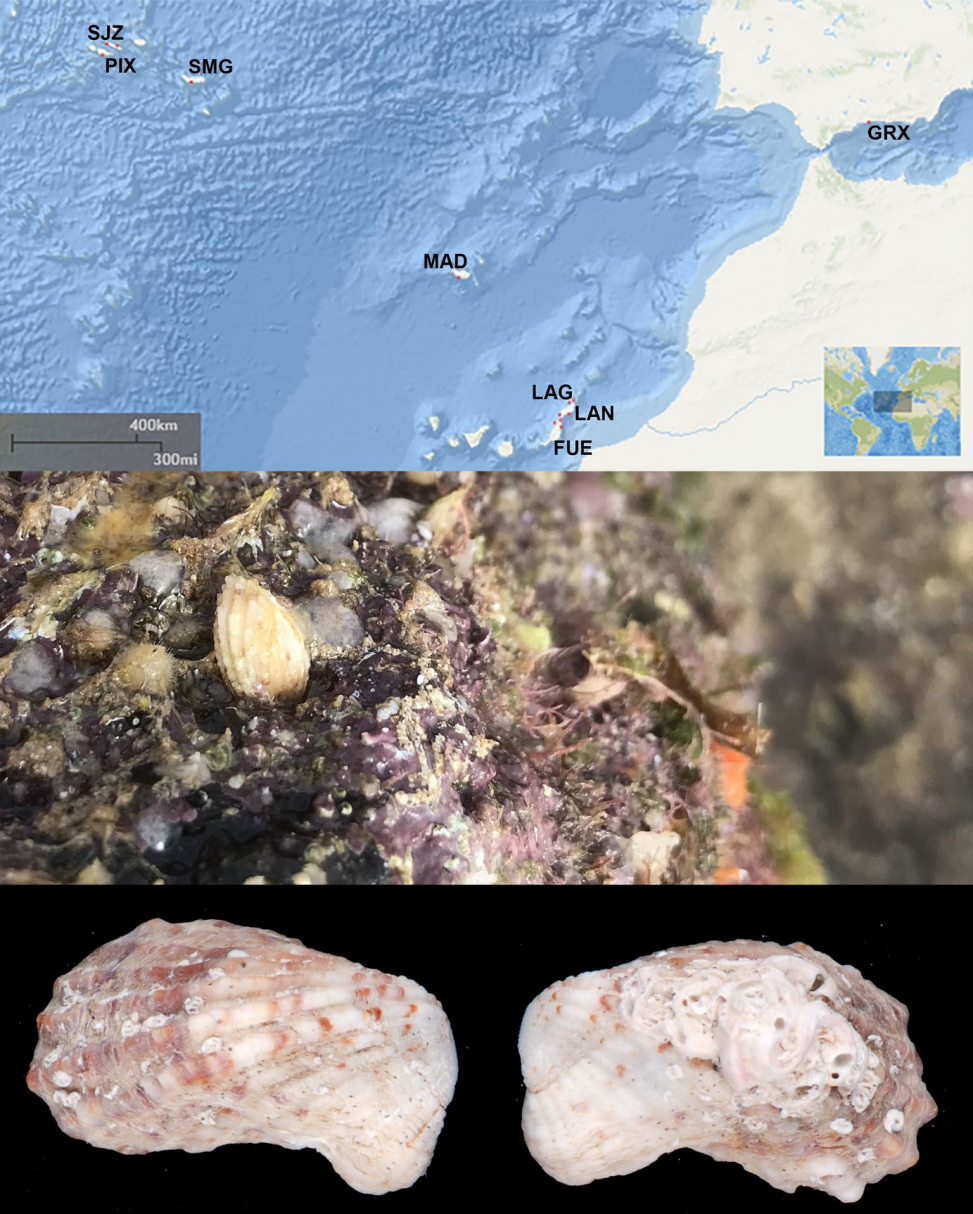
(**A**) Study area in the Northeast Atlantic Ocean with indication of sampling locations (red dots). Geographical location of the Azores Archipelago (Portugal) and the sampled islands of São Jorge (SJZ), Pico (PIX) and São Miguel (SMG); Madeira archipelago with only one sampled location (MAD); Canary Islands with sampling locations in La Graciosa (LAG), Lanzarote (LAN) and Fuerteventura (FUE); Mediterranean Sea with sampling location in Granada province (GRX). General Bathymetric Chart of the Oceans (GEBCO) Map derived from NOAA/NCEI Bathymetric Data Viewer by NOAA/NCEI https://www.ncei.noaa.gov/maps/bathymetry/. *(***B**) Intertidal boulder with small holes whereby specimens of the bivalve *C*. *calyculata* were mostly found. (**C**-**D**) beached shells of *Cardita calyculata* collected at Praia da Vitória, Terceira Island, Azores (Frias Martins collection, FM-24). (**C**) Right valve, dorsal view. (**D**): Left valve, dorsal view. Length: 17 mm

A total of 165 *Cardita calyculata* specimens, from 13 locations in the archipelagos of the Azores, Madeira and Canary Islands as well as the Mediterranean Sea were used in this study (Fig. 1; Table 1). They were either collected directly from the intertidal habitats or retrieved from collections from the Department of Biology of the University of the Azores (DBUA). Fresh samples were found within scraped algal turf (Velas, São Jorge) or attached via their byssus thread to interstitial holes in the rocky shores and under boulders (all other locations). The whole individual was collected and stored in 96% ethanol until DNA isolation. Permits for sampling were issued by the respective authorities. Samples from Câmara dos Lobos (Madeira: DBUA 1585) and Lagoa (São Miguel Island, Azores: DBUA 1799) were provided by the DBUA marine molluscs’ collection.

**Table 1:** Geographical location (decimal degrees) and population sizes (n) of analysed *Cardita calyculata* populations

*N*	Location	Coordinates
14	Caletòn, Granada (GRX), Spain	36.744021, -3.605433
27	Velas, São Jorge (SJZ), Azores (AZ)	38.677780, -28.208623
1	Fajã Santo Cristo, São Jorge (SJZ), Azores	38.626957, -27.933348
25	Lajes do Pico, Pico (PIX), Azores (AZ)	38.395019, -28.256295
2	Salinas Câmara de Lobos, Madeira (MAD)	32.646203, -16.973527
9	Playa de la Cocina, La Graciosa (LAG), Canary Islands (CI)	29.220244, -13.543317
1	Playa Lambra, La Graciosa (LAG), Canary Islands (CI)	29.279393, -13.495312
18	Playa Quemada, Lanzarote (LAN), Canary Islands (CI)	28.907084, -13.735678
21	Pechiguera, Lanzarote (LAN), Canary Islands (CI)	28.855567, -13.873209
7	Bajas de Bristol, Fuerteventura (FUE), Canary Islands	28.744395, -13.869549
20	Playa del Hierro, Fuerteventura (FUE), Canary Islands (CI)	28.739033, -13.952371
13	Los Lapios, Fuerteventura (FUE), Canary Islands (CI)	28.545822, -13.831355
7	Playa de la Barra, Fuerteventura (FUE), Canary Islands (CI)	28.699834, -14.017138

### DNA extraction protocol

Total genomic DNA (gDNA) was extracted from either the entire animal removed from the shell (when the specimen was smaller than 2 mm) or from a muscle fragment. A SDS based buffer tissue protocol was used for DNA extraction, explained in the Supplementary Materials. An electrophoretic run in agarose gel 1.5% at 80 V for 30 min was performed to evaluate DNA integrity in comparison to a length marker. Clear bands larger than about 10,000 bp were considered as successfully extracted and used in the further steps of PCR amplification. Most bands were larger than 23,000 bp.

### Microsatellite analysis

#### SSR-GBAS marker development

A low coverage Illumina MiSeq run from one *C. calyculata* individual (CC18 from Lajes do Pico, Pico Island, Azores) was conducted for marker development and raw reads can be accessed through Genbank, BioProject ID: PRJNA1099938. Library preparation and shot-gun sequencing on an Illumina MiSeq paired-end (PE) 300 bp run were performed as a service at the Genomics Service Unit, Ludwig-Maximilian University Munich, Germany. The protocol described by Curto et al. [51], was used to identify sequences containing SSR-GBAS. Such protocol is implemented by the software FastQC v0.11.9 [53], Trimmomatic v0.39 [54], Usearch v11 [55] and a final script SSR_pipeline’s script SSR_search.py [56], after which primers were designed through Geneious v2022.2.2 [57]. Details including PCR primer testing can be found in Supplementary Material (*Laboratorial procedures for Illumina sequencing* and *SSR-GBAS marker development*). Thirty-six out of the initially designed 50 primer pairs successfully generated amplicons within the expected amplicon size and were combined in three multiplex primer mixes containing 12 primer pairs, with each primer at a final concentration of 1 µM (Table S1, Supplementary Material).

#### Multiplex PCR and illumina sequencing

Multiplex amplification was conducted in PCR reactions of 5 µl: 2.5 µL of QIAGEN Multiplex PCR Master Mix (Qiagen, CA, USA), 0.5 µL of each primer mix, and 2 µL of gDNA diluted in 1:4 proportion, following the same cycling conditions as the single PCR to test the primers individually. The remaining library preparation steps included PCR cleanup with AMPure XP beads (Beckman Coulter Inc., USA) and indexing, following the protocol from Curto et al. [52]. The procedure resulted in one amplicon library per individual characterised by a unique dual index combination. The resulting libraries were pooled equimolarly and sent for Illumina MiSeq PE 300 bp sequencing as a service at the Genomics Service Unit at Ludwig Maximillian Universität (München, Germany). Raw reads can be accessed through Genbank, BioProject ID: PRJNA1099938.

#### SSR data analysis

Raw FastQ sequence data of each sample was automatically recognized and extracted by the MiSeq equipment based on the unique index combinations. Quality control and merging steps with FastQC v0.11.9 was used for control of results and conducted as described above, and Trimmomatic v0.39 and Usearch v11 were used as part of the Script and for the genotype call. The SSR_GBS_pipeline scripts are available at GitHub (https://github.com/mcurto/SSR-GBS-pipeline). Hereby, plots were generated from frequency distribution of sequence length in the library highlighting the ones used for the subsequent allele calling step, allowing for manual verification of marker duplication or other potential errors. All final allele call using WAI dataset resulted in a codominant matrix for further population genetic analyses. Genotyping based on sequence length is used as an intermediate step to filter out sequences resulting from amplification and sequencing artifacts. The resulting sequences are then used to produce a consensus and define potential variants within each length. This results in up to two sequences per individual (considering the organism is diploid). Allele numbers are then attributed to each unique sequence per marker to make codominant genotypes. In such way, variation from the repetition motif and from the flanking regions are combined.

#### Quantifying genetic structure from genotype (SSR-GBAS) data

Samples with more than 50% missing data were removed from the analysis; the same was done for markers with more than 60% missing data. Such high cutoff values were established to keep a higher sample representation. The high prevalence of allele dropout and missing data throughout the nuclear loci of invertebrate and more specifically molluscs’ species has often been reported as an issue for data analysis. Conservative cutoffs have been reported to the loss of biological meaningful information (e.g [58]). GenAlx v6.5 [59] allowed the measurements of deviations from Hardy-Weinberg Equilibrium (HWE), number of alleles (Na) and effective (Ne) alleles (number of equally frequent alleles needed to achieve the expected heterozygosity of the studied population), Shannon’s Information Index (I), observed (*Ho*) and expected (*He*) heterozygosity and population inbreeding coefficient (FIS). Total number of alleles was calculated per locus and FreeNa [60] was used to quantify null alleles per population, considering 100 replicates. The limit for inferring a significant amount of null allele frequencies per locus was set to 0.2 [61–63].

A hierarchical analysis of molecular variance (AMOVA) was performed in GenAlEx to test the degree of differentiation between localities and regions (different archipelagos and the mainland population). Pairwise FST divergence between populations was calculated to assess evolutionary divergence between the populations. The above-mentioned genetic diversity analyses were done for populations with more than 5 individuals (Fajã de Santo Cristo, São Jorge, Azores; Playa Lambra, La Graciosa, the Canary Islands; and Salinas Câmara de Lobos, Madeira were excluded).

Patterns of genetic distances between all individuals were inferred in a Principal Coordinate Analysis (PCoA). The software STRUCTURE v2.3.4 [64] was used to produce a probability of assignment of each individual to a hypothetical group with assumptions of HWE. With the number of clusters (K) varying between 1 and 13, STRUCTURE ran for 10 independent replicates for 100,000 generations, following a burn-in period of 100,000 (default settings were maintained for the admixture model and correlated allele frequencies). The results from STRUCTURE across the K-values (optimal inferred by Evanno et al. method; [65]) were summarized and graphically displayed resorting to the online pipeline CLUMPAK [66]. Such analysis was done for all populations together and then just for the Canary Islands populations.

Geographical information was included in the inference of genetic structure using GeneLand v. 4.0.6 [67]. This method uses georeferenced individual multilocus genotypes to infer the number of populations and the spatial location of genetic discontinuities between those populations [67]. Moreover, this method does not follow the expectation of HWE addressing the fact that some populations showed significant deviations. Such analysis was done both for all samples together and then separately for the Canary Islands. MCMC simulation parameters for both GenLand analyses were set as follows: Ploidy = diploid; number of populations = 13, number of iterations = 100,000; thinning = 100, allele frequency model = correlated; spatial model = TRUE; null allele model = TRUE; burnin = 200.

BayesAss edition 3 (BA3) was used to infer migration rates between populations. The program ran 50,000,000 generations after a burning period of 25,000,000. Mixing parameters were adjusted to reach a final acceptance rate between 20% and 60% resulting from the following values: allele frequencies (--deltaA) 0.4, inbreeding coefficient (--deltaF) 0.4 and migration rates (--deltaM) 0.2. MCMC parameter convergence was monitored using the program Tracer v1.7.1 [68].

### Mitochondrial analysis

The COI region was amplified with the primers jgLCO1490/jgHCO2198 [69] in either 20 µL or 10 µL reaction solutions to accommodate the specifications of different sequencing services: the commercial facility AGENTA GeneWiz (Leipzig, Germany) and the Centre for Molecular Analyses (CTM from CIBIO-InBIO Research Centre, Vairão, Portugal), respectively. Details about the PCR reactions, cycling conditions, and Sanger sequencing of the COI dataset can be found in Supplementary Materials.

The resulting COI chromatograms were manually checked for the presence of misreads with Geneious Prime v2022.2.2. Presence of premature stop codons was inspected through AliView [70]. All the sequences generated in this study were deposited in GenBank (accession numbers, CC7: PP583649; CC9: PP583650; CC361: PP583627; CC239: PP583628; CC447: PP583629;

CC393: PP583630; CC391: PP583631). The COI dataset was aligned with Geneious Prime using global alignment with free end gaps and a cost matrix of 65% similarity. Sequences under 300 bp were excluded from the study and the remaining were trimmed to the overlapping length (406 bp).

Population structure was visualized through a haplotype network analysis, inferred through the parsimony approach with 95% connection limit implemented in TCS software [71]. The output was rendered using the web-based program tcsBU [72], which allows the integration of the geographic location of each sample within the TCS results. Raw (p) distances amongst *C. calyculata* haplotypes were calculated with MEGA v11.0.13 [73] to detect the presence of potential cryptic species by measuring the evolutionary divergence between sequence pairs.

The COI was also used to infer the phylogenetic relationships among *C. calyculata* populations. COI sequences of *C. calyculata* from other locations in the Mediterranean deposited in GenBank were added to the dataset (Table S2, Supplementary Material), as well as another species from the Mediterranean – *Cardita variegata* (Bruguière, 1792; GenBank: GQ166578) – to serve as an outgroup for the phylogenetic analyses. Sequences were collapsed into haplotypes using the web-based program Alignment Information Environment (ALTER [74]), . Jmodeltest v2.1.9 [75, 76] was used to determine the best substitution model under the Akaike Information Criterion (AIC). A maximum likelihood tree was constructed with Raxml-ng v1.2.1 [77]. Transfer Bootstrap Expectation (TBE) values were used to infer branch support. Such values have been shown to provide better robustness and repeatability for deep branch analyses when compared to Felsenstein’s bootstrap proportions (FBPs [78]), . Fig Tree v1.4.4 was then used to visualize the resulting tree.

## Results

### SSR-GBAS marker development

For the SSR-GBAS marker discovery, the MiSeq run produced a total of 7,532,890 reads, 3,514,210 of which passed the quality control and merging steps. Of these, 22,536, 10,233, 32,751 and 4,369 contained di, tri, tetra and penta-nucleotide repeats, respectively.

#### Genetic diversity measures

The MiSeq run for SSR-GBAS produced 9,645,090 raw paired reads from which 5,517,442 passed the quality control and multiplex steps and were used to call genotypes. The resulting matrix contained 21 SSR-GBAS with less than 60% missing data (Table S3, Supplementary Materials). Deviations from HWE were detected for most loci (‘loci deviating from HWE’ in Table 2). However, none deviated consistently across all populations. All populations showed an excess of homozygotes and hence positive inbreeding coefficient (FIS, Table 2). The populations with the highest genetic diversity values (considering the resulting N, Na, Ne, I, Ho and uHe) were: Velas (São Jorge Island, Azores); secondly Playa el Hierro (Fuerteventura Island, the Canary Islands) and lastly Pechiguera (Lanzarote, the Canary Islands) (Table 2). A mean of 35 alleles per locus was calculated and FreeNa showed a mean of 7 loci per population with a higher than 2% frequency of null alleles, excluding populations from Lajes (Pico Island, Azores) which showed just 2 loci with such frequency (Table S.4). Pairwise FST divergence between populations showed greater distances between the mainland (Caletón, Granada) and the sampled NE Atlantic Archipelagos of the Azores and the Canary Islands (mean of 0.34; Table 3).

**Table 2:** Genetic diversity measures for the SSR-GBAS markers for each population of *Cardita calyculata*: sample Size/analysed loci (N), no. Alleles (na), no. Effective alleles (ne), Shannon’s Information Index (I), observed heterozygosity (Ho) and unbiased expected heterozygosity (uHe), inbreeding coefficient (FIS) and number of loci deviating from hardy-Weinberg equilibrium (HWE) out of 21 overall loci. GRX: Granada, Spain. PIX: Pico Island, Azores. SJZ: São Jorge Island, Azores. SMG: São Miguel Island, Azores. FUE: Fuerteventura, Canary Islands. LAN: Lanzarote, Canary Islands. LAG: La Graciosa, Canary Islands. Mean and standard errors (SE) values over the 22 loci are presented

Population		*N*	Na	Ne	I	Ho	uHe	FIS	loci deviating from HWE
Càleton, GRX	Mean	8.905	4.524	2.844	1.008	0.323	0.508	0.339	9
	SE	1.244	0.833	0.559	0.166	0.076	0.073	0.103	
Velas, SJZ	Mean	**24.476**	**9.619**	**4.255**	**1.578**	**0.494**	**0.677**	0.284	14
	SE	1.156	0.829	0.501	0.141	0.067	0.051	0.082	
Lajes do Pico, PIX	Mean	**21.429**	7.714	3.673	1.360	**0.494**	0.613	0.171	8
	SE	1.146	0.903	0.474	0.154	0.072	0.062	0.071	
Playa de la Cocina, LAG	Mean	6.762	4.524	3.004	1.176	0.318	0.647	**0.475**	13
	SE	0.581	0.505	0.284	0.117	0.060	0.055	0.084	
Playa Quemada, LAN	Mean	13.381	**7.286**	**4.410**	1.413	0.397	0.645	0.367	14
	SE	1.245	1.143	0.755	0.176	0.061	0.064	0.068	
Pechiguera, LAN	Mean	**15.905**	7.048	4.270	**1.441**	0.370	**0.666**	**0.468**	15
	SE	1.426	0.832	0.598	0.153	0.057	0.059	0.062	
Bajas de Bristol, FUE	Mean	4.952	4.429	3.395	1.148	0.374	0.619	0.332	3
	SE	0.455	0.563	0.449	0.153	0.071	0.075	0.091	
Playa del Hierro, FUE	Mean	14.429	**7.905**	**4.715**	**1.547**	**0.425**	**0.701**	0.361	9
	SE	1.362	1.021	0.719	0.159	0.068	0.057	0.083	
Los Lapios, FUE	Mean	9.143	5.762	3.855	1.265	0.423	0.603	0.294	10
	SE	0.874	0.752	0.691	0.166	0.071	0.069	0.072	
Playa de la Barra, FUE	Mean	4.952	4.143	3.172	1.133	0.360	0.653	**0.445**	8
	SE	0.455	0.489	0.399	0.127	0.076	0.059	0.100	

**Table 3:**
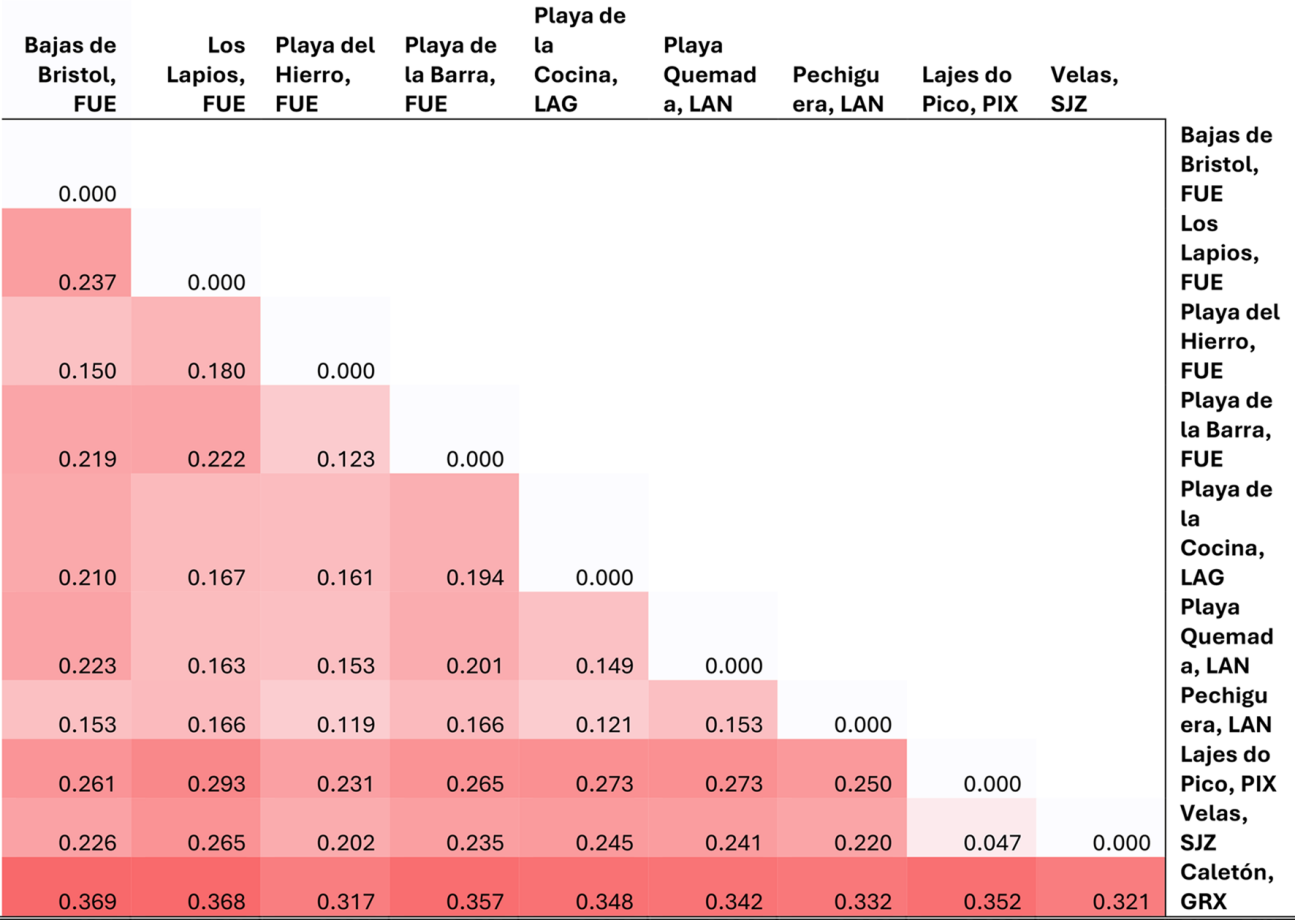
Pairwise FST divergence between the sampled *Cardita calyculata* populations. Heat maps darker red correlates with higher distance values. For other abbreviations, see table 2

#### Genetic and structure analyses

**Fig. 2:**
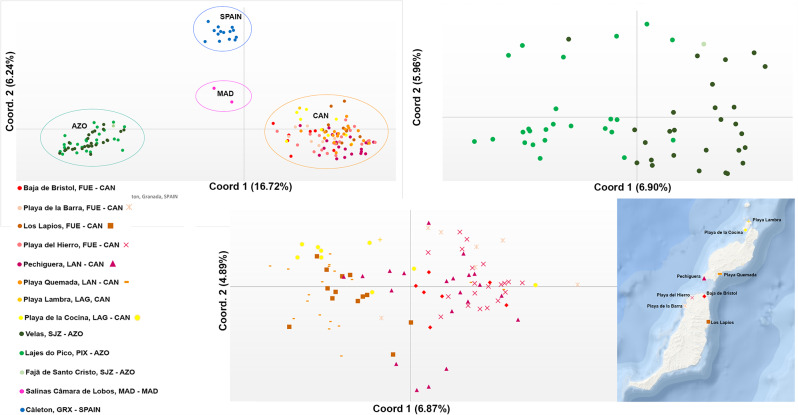
Principal coordinate analysis of *Cardita calyculata* and % of variance explained. PCoAs conducted as implemented in GenAlEx v6.5; populations in study are coded by colour. AZO: Azores Archipelago. CAN: Canary Islands. MAD: Madeira Archipelago. (**A**) All locations. (**B**) Azores. (**C**) Canaries (with relative map of sampled locations)

The PCoA revealed a clear distinction between the Mediterranean (Granada Province), the Azores (São Jorge and Pico islands), Canary Islands (La Graciosa, Lanzarote and Fuerteventura islands), and Madeira (Porto Santo; Fig. 2A). When the patterns were analysed per archipelago, some degree of structuring can be found between Pico and São Jorge (Fig. 2B). The similarity of *C. calyculata* from Fajã do Santo Cristo (São Jorge Island) with other Azorean localities could not be assessed, as only one individual represented this population in the current dataset. In the Canary Islands, populations of Playa Quemada (Lanzarote), Los Lapios (Fuerteventura) and to some degree Playa de la Cocina (La Graciosa) clustered closer together, distancing from the group formed by Playa del Hierro (Fuerteventura), Pechiguera (Lanzarote), Playa de la Barra (Fuerteventura) and Bajas de Bristol (Fuerteventura) (Fig. 2C).

**Fig. 3:**
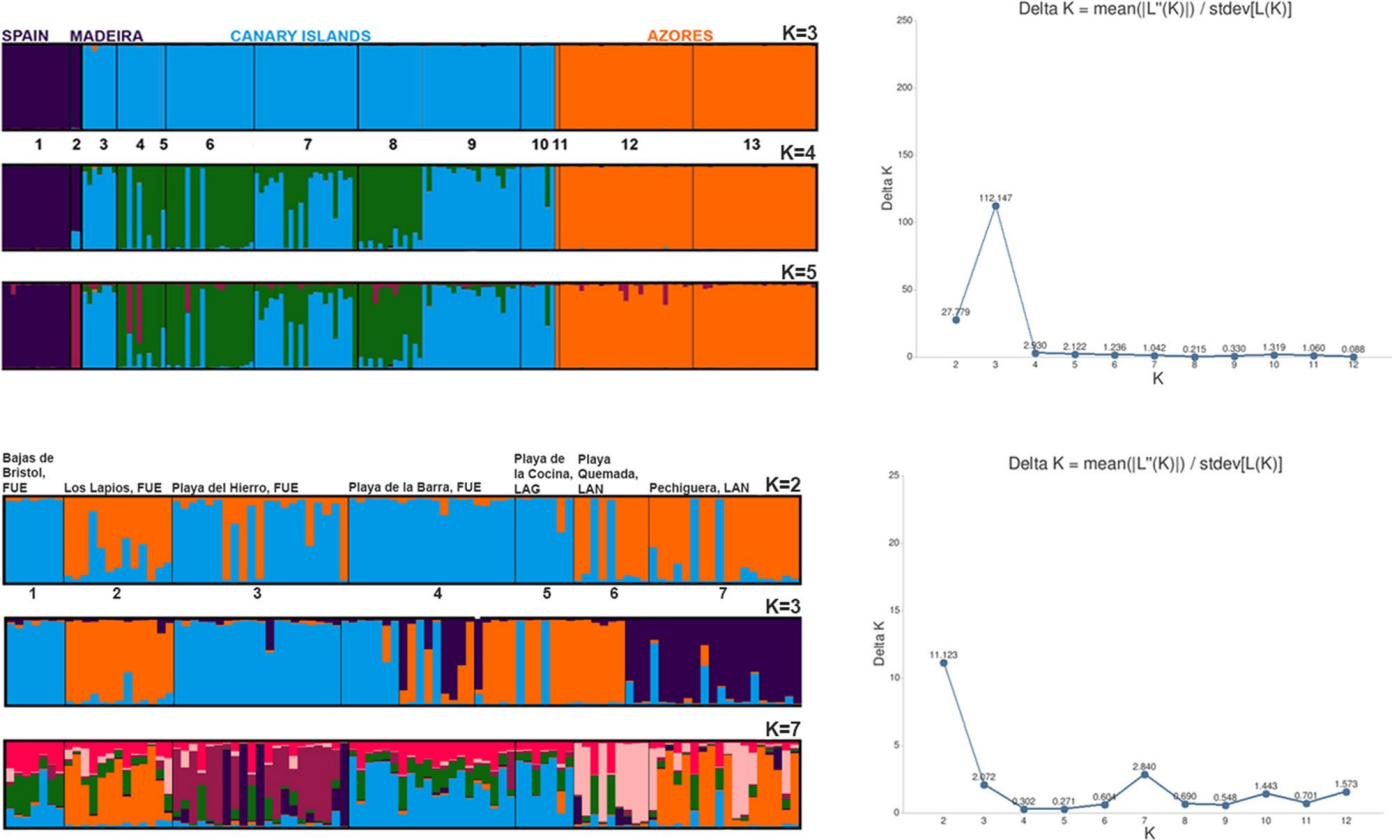
STRUCTURE analysis based on the allele frequencies of all samples. Each colour represents a ‘*K population*’ inferred by STRUCTURE, the variation of which correlates with the population membership of each individual (**A**, **B**). (**A**) The 13 analysed populations divided into sections :1 - Caletón, Granada, Spain; 2 - Salinas Câmara de Lobos, Madeira Island; 3 - Bajas de Bristol, Fuerteventura, Canary Island; 4 - Los Lapios, Fuerteventura, Canary Island; 5 - Pechiguera, Lanzarote, Canary Island; 6 - Playa del Hierro, Fuerteventura, Canary Island; 7 - Playa de la Barra, Fuerteventura, Canary Island; 8 - Playa de la Cocina, La Graciosa, Canary Island; 9 - Playa Lambra, La Graciosa, Canary Island; 10 - Playa Quemada, Lanzarote, Canary Island; 11 - Fajã do Santo Cristo, São Jorge Island, Azores; 12 – Lajes do Pico, Pico Island, Azores; 13 - Velas, São Jorge Island, Azores. B: Delta K values; K = 3 “Best” K according to Evanno’s deltaK. C: Canarian populations divided into 7 sections: 1 - Bajas de Bristol, Fuerteventura; 2 - Los Lapios, Fuerteventura; 3 - Playa del Hierro, Fuerteventura; 4 - Playa de la Barra, Fuerteventura; 5 - Playa de la Cocina, La Graciosa; 6 - Playa Quemada, Lanzarote; 7 - Pechiguera, Lanzarote. D: Delta K values; K = 2 “Best” K according to Evanno’s deltaK, for the Canary Islands

**Fig. 4:**
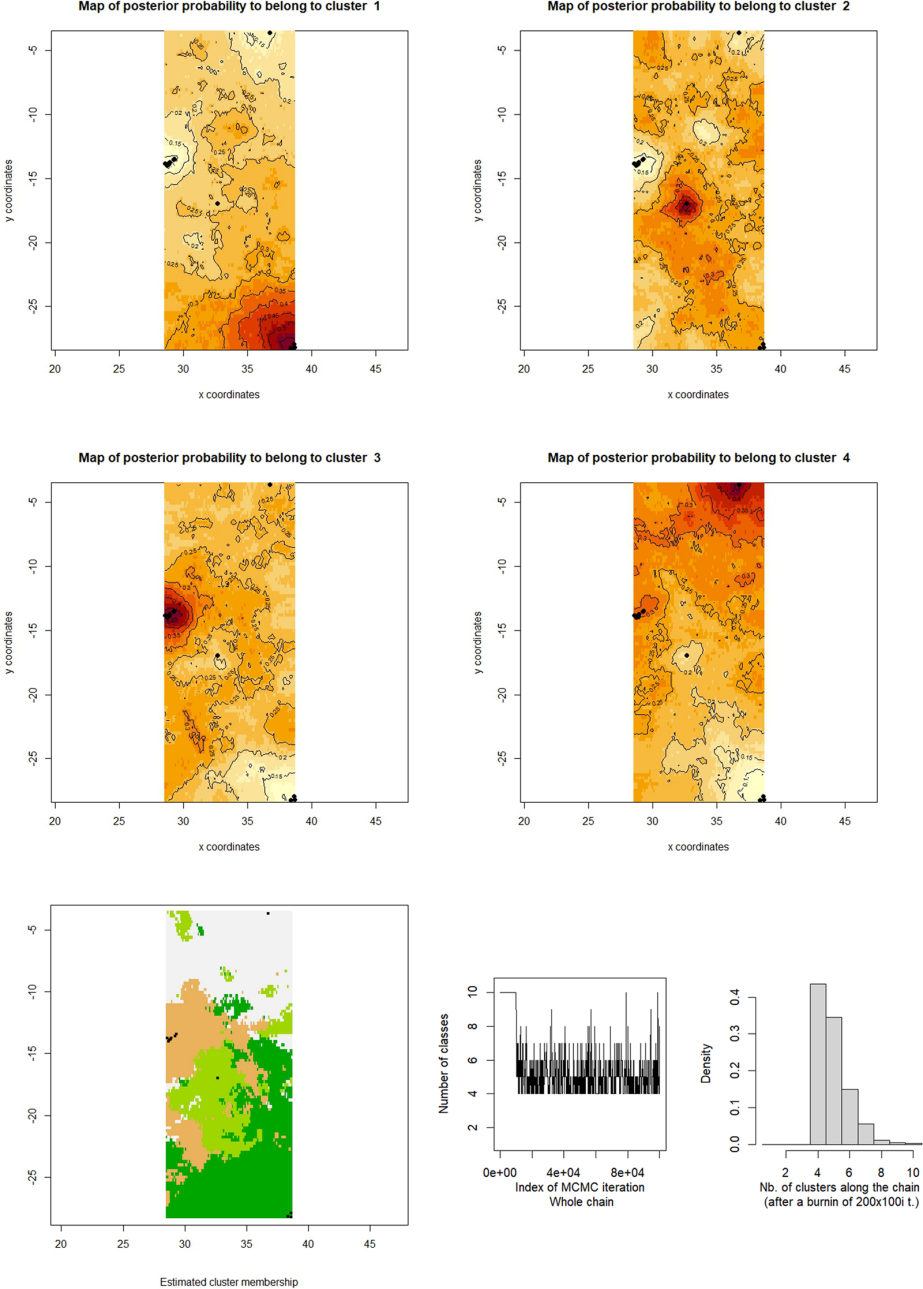
Geneland population clustering across space derived from SSR-GBAS data of all *C. calyculata* samples. (**A**) Map of posterior probabilities of population membership. The spatial location of genetic discontinuities are provided (i.e. genetic boundary between the two populations). Each panel shows Geneland maps of the study area with the relative posterior probability of belonging to the 4 different clusters (K = 4 inferred as optimal). Black dots represent the geographical position of the sampled locations. The lighter colouration reflects a higher posterior probability of membership to the different clusters whilst the darker reflects the lowest. (**B**) Synthetic map of the mode of the posterior probability distribution for each pixel belonging to each inferred population. Black dots represent the geographical position of the sampled locations. (**C**) Trace of number of populations along the MCMC run with variable number of classes and histogram of simulated values. This run displays a clear mode at K = 4 which is hence the maximum a posteriori estimate of K

The AMOVA analysis (Table 4) revealed that 25% of the variation was explained by divergence among regions (Azores, Canary Islands and Mediterranean) and only 5% between populations within regions. The STRUCTURE analysis revealed an optimal delta-K of 3 for all analysed populations in the NE Atlantic (Fig. 3B). The three groups inferred separate the cluster of Mediterranean and Madeira from another formed by the Azores and the Canary Islands (Fig. 3A). The GeneLand analysis of all samples suggested an optimum clustering of 4 groups: Canary Islands, Madeira, Azores and Mediterranean (Fig. 4).

**Table 4:** Hierarchical analysis of Molecular Variance (AMOVA) among *Cardita calyculata.* Analysis performed among populations and among geographical regions (Canary Islands, Azores, Mediterranean), localities and individuals with 999 permutations

Source	df	SS	MS	Est. Var	%
Among regions	2	482.661	241.330	2.439	25
Among populations	7	179.893	25.699	0.526	5
Among individuals	151	1496.934	9.913	3.042	31
Within individuals	161	616.500	3.829	3.829	39
Total	321	2775.988		9.836	100

**Fig. 5:**
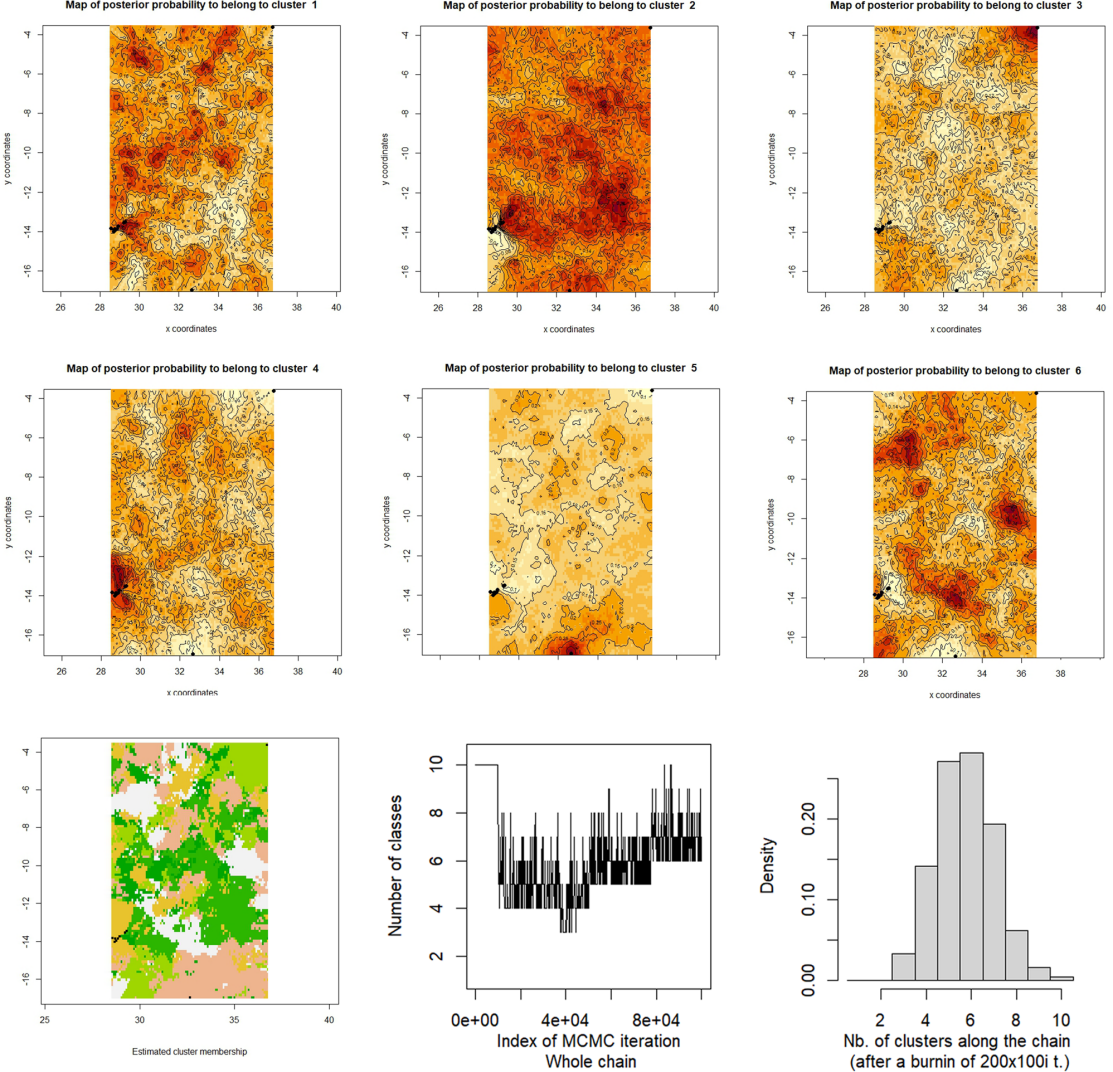
Geneland population clustering across space derived from SSR-GBAS data of *C. calyculata* samples from the Canary Islands. (**A**) Map of posterior probabilities of population membership. The spatial location of genetic discontinuities are provided (i.e. genetic boundary between the two populations). Each panel shows Geneland maps of the study area with the relative posterior probability of belonging to the 6 different clusters (K = 6 inferred as optimal). Black dots represent the geographical position of the sampled locations. The lighter colouration reflects a higher posterior probability of membership to the different clusters whilst the darker reflects the lowest. (**B**) Synthetic map of the mode of the posterior probability distribution for each pixel belonging to each inferred population. Black dots represent the geographical position of the sampled locations. (**C**) Trace of number of populations along the MCMC run with variable number of classes and histogram of simulated values. This run displays a clear mode at K = 6 which is hence the maximum a posteriori estimate of K

STRUCTURE analysis for just the Canary Islands revealed an optimal delta-K of 2 (Fig. 3D). The groups inferred from the analysis suggest an east-west differentiation, with La Graciosa and Lanzarote grouping closer together (Fig. 3C). The GeneLand analysis of the Canarian samples shows 5–6 optimum clusters (Fig. 5), being the localities Los Lapios mostly assigned to cluster 5, Playa de la Cocina to cluster 3 and the remaining to clusters 1 and 4.

BayesAss analysis (Table 5) showed Pechiguera (Lanzarote) and Playa del Hierro (Fuerteventura) to be the more frequent sources of migrants for populations in the Canary Islands. The former location appeared to comprise 17% of the genetic pool of Playa Quemada (Lanzarote), 17% of Los Lapios (Fuerteventura) and 12% Playa de la Cocina (La Graciosa). For Azores, populations seem to be migrating mostly from Velas (São Jorge) to Lajes do Pico (Pico Island), the former comprising 11% of the genetic pool of the latter.

**Table 5:**
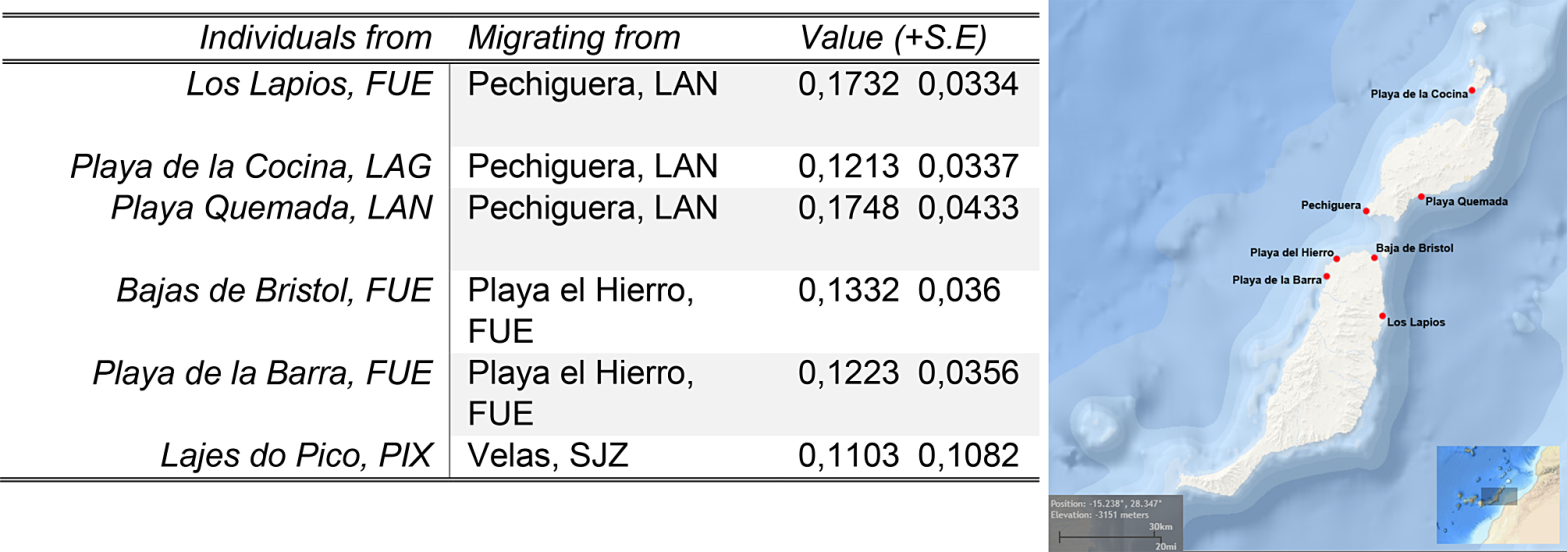
Estimates of migration rates retrieved from multilocus genotype data of *Cardita calyculata* populations through BayesAss v3. Values reflect the proportion of individuals of the sink population that originate from the source. Only values greater than 0.05 are reported. Map on the right shows locations of the relevant populations in La Graciosa, Lanzarote and Fuerteventura (Canary Islands). Map derived from NOAA/NCEI Bathymetric Data Viewer by NOAA/NCEI https://www.ncei.noaa.gov/maps/bathymetry/. For other abbreviations, see table 2

### mtDNA COI data

**Fig. 6:**
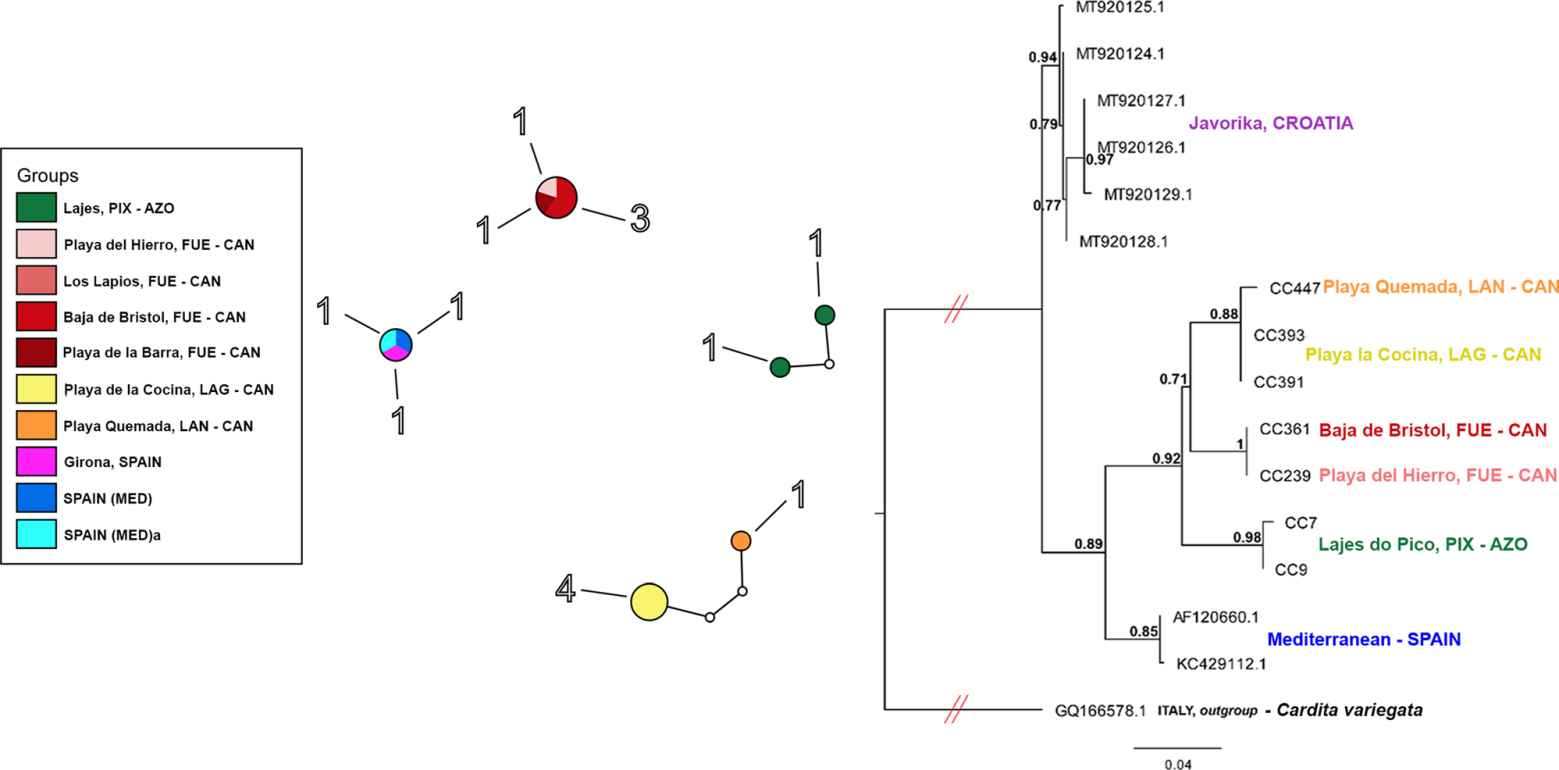
Graphical representation and phylogenetic relationship of *Cardita calyculata* from the NE Atlantic and Mediterranean. (**A**) Haplotypic visual representation of 11 colour-coded populations and 15 COI sequences, at 95% parsimony connection limit. Absolute haplotype frequency presented nearby each circle, which vary in size in relation to the frequency; each uncoloured small circle in the lines connecting haplotypes represents a single mutational change. Haplotype diagram obtained with TCS v1.21. (**B**) Maximum likelihood tree (Raxml-ng) of 16 COI sequences of Cardita, considering 1000 replicates for the TBE bootstrap inference

The COI dataset comprised of a total of 22 sequences (12 original sequences plus 10 from GenBank) from the Mediterranean, Canaries and Azores (Table S2, Supplementary Materials). GenBank sequences from Croatia (Mediterranean) were excluded from the TCS analysis as the further clustering of these samples was not considered relevant for the current study. Amongst the COI dataset, four main clusters were distinguished with little to no divergence amongst the haplotypes: two from the Canaries (one represented by populations from Fuerteventura and the other by Lanzarote and La Graciosa), one from Mediterranean Spain and one from the Azores (Fig. 6A). No haplotypes were shared between these clusters, however the number of individuals per archipelago in the COI dataset was low ((only two individuals from Pico represent the Azores, compared to five individuals from different islands in the Canary Islands), thus conclusions about haplotypes shared between populations have to be drawn with caution.

The best substitution model detected by JmodelTest and used in the Raxml analysis was TVM + I. The tree was rooted on a closely related species, *Cardita variegata* (GenBank: GQ166578.1). Bootstrap TBE values showed relatively high support for branch splitting (> 71%). When looking at the Atlantic populations, the Azorean group (represented by Pico) appeared as a sister group to the Canary Islands. Its divergence to the Canarian populations seemed to be similar to that between the two clades within the Canary Islands (one represented by Lanzarote and La Graciosa, and the other represented by Fuerteventura; Fig. 6B). The mean interspecific p-distance detected in MEGA v11 is 15%. Differences between Macaronesian samples and other sites ranged from 0% [between Playa del Hierro and Bajas de Bristol (Fuerteventura, Canary Islands)] to 8% [between Lajes do Pico (Pico, Azores) and Javorika (Croatia, Mediterranean)] (Table S5, Supplementary Materials).

## Discussion

### Population structure

The data obtained is congruent with the existence of barriers to gene flow between and within the archipelagos. As within the ocean system any physical/geological feature according to classical definitions does not represent a barrier to geneflow due to the inherent connectivity of the aquatic medium; the genetic structure and differentiation found result from the evolutionary interaction between *C. calyculata* life-history traits, such as its dispersal ability, density-dependent effects and the oceanographic environment set through the varying geological and climatic shifts. The nature of such interaction and its consequent implications on the population genetic patterns of *C. calyculata* are hereafter discussed.

The analysis of the mitochondrial COI supports the genetic differentiation between the Mediterranean, the Azores and the Canary Islands. Pico (Azores) forms a unique haplotype whilst the Canary Islands are characterized by a diverse array of haplotypes. Within this Archipelago, La Graciosa and Lanzarote appear closely related whilst Fuerteventura yields a unique haplotype (Fig. 6A). Such populational differentiation is reflected in the SSR-GBAS structure, whereby four groups (Mediterranean, Azores, Madeira and Canary Islands) diverge (Fig. 2A). This inference is also supported by the different clustering analyses and the AMOVA analysis (25% of the variation is explained among regions).

Further genotype structure and increased diversity are found in the Canary Islands. Only the eastern islands of the Archipelago were sampled, thus generalized conclusions over the entire Canarian Archipelago cannot be outlined. Genetic structure is present in Lanzarote, La Graciosa and Fuerteventura islands, which formed a single landmass during the Last Glacial Maximum (LGM) [79]. For the Canary Islands, once the SSR-GBAS diversification is displayed spatially (Figs. 2C and 3D), individuals from Los Lapios (Fuerteventura) and Playa Quemada (Lanzarote) and, to some degree, Bajas de Bristol (Fuerteventura), are more similar to each other when compared to the group formed by Playa de la Barra, Playa del Hierro (Fuerteventura) and Pechiguera (Lanzarote). The first group situated across the west coast of both islands, the second group at the east coast. Playa de la Cocina (La Graciosa) is taking an intermediate position between the groups. Such a pattern is congruent with an east-west dispersal barrier which might have restricted gene flow through time and enabled the changes in allele frequencies. The pattern can thus be an imprint from the connection between the islands during LGM.

For the Azorean islands only a low amount of reference populations was available. DNA extracted from specimens from Flores and São Miguel was poor in quality, likely attributable to the age of the samples dating back to 2019, in contrast to the samples from São Jorge and Pico, for which DNA extraction took place within a month from collection. Additionally, no specimens were found along the shores of Santa Maria, leading to a low representation of locations in the Azorean Archipelago when compared to the Canaries. The successfully analysed populations exhibit patterns similar to the Canary Islands, and both populations can be differentiated in the PCoA reflecting the geographical distance between the two islands Pico and São Jorge, where they were sampled.

Genetic differentiation values suggest Madeira and Granada (Mediterranean) populations to be mostly distant from the remaining populations analysed. In the PCoA, they appear in an intermediate position between the archipelagos and the mainland. However, no definite assumptions can be made for Madeira population, as only three individuals were successfully analysed. A genetic ‘bridge’ between the mainland and the different regions of the north-east Atlantic can be outlined for Madeira by the structure analysis with an intermediate cluster assignment at K4 and K5 of Madeira individuals.

The low percentage of diversity explained by the two coordinates in the PCoA might result from the high diversity and evolutionary divergence of the genotype data within and between the sampled archipelagos. The positive FIS values in the dataset (cf. Table 2) suggest deviations from HWE which might be a consequence of inbreeding, Walhund effect or null alleles. As stated above, *C. calyculata* shows consistent morphological features suggesting a brooding reproductive strategy. Such a life-history adaptation contextualized within the varying oceanographic conditions throughout its geological lifetime, can generate high levels of homozygosis due to the added influence of inbreeding and Wahlund effect. High levels of genetic drift and inbreeding have been reported for mollusc species, whereby cases of functional hermaphroditism and self-fertilization, especially in bivalve species, could have been evolved as efficient reproductive strategy for species with low population densities [80]. Although the analysis of the specific reproductive strategy of *C. calyculata* surpasses the aim of the present study, the species displays a population genetic structure in accordance with the above-described patterns, suggesting the potential interplay of such mechanisms in influencing the overall population diversity patterns.

Amongst bivalves specifically, null alleles can appear with an unusually high frequency in microsatellite analysis [81, 82] as large population sizes often lead to a high number of alleles present in a population with high in-between allelic divergences (mean of 35 alleles per locus was found in this study, Table S4, Supplementary Material). By reducing the genetic diversity between populations (F and genetic distance values generally increase with decreasing within-population genetic diversity), null alleles may underestimate population differentiation [61, 83] since divergent alleles are more likely to drop out. Nonetheless, simulation studies suggest that null allele frequencies up to 8% have minor effects on classical estimates of population differentiation, but that higher frequencies would bias such parameters [61, 63]. We thus suggest that the genetic pattern of populations of *C. calyculata* still reliably represents their differentiation in the NE Atlantic, potentially being more pronounced than what is observed.

Overall, it appears that *C. calyculata* shows a relatively high degree of differentiation between regions in the northeast Atlantic. Although such patterns cannot be generalized for marine benthic invertebrates, similar genetic differentiation structures have been found in other sessile non-planktotrophic marine organisms [13, 84]. By analysing the phylogeographic relationships amongst larva-lacking peracariid crustaceans inhabiting the intertidal shores of Macaronesia, Vieira et al. [84], demonstrated that the isopod *Dynamene edwardsi* (Lucas, 1849) comprised multiple, deeply divergent evolutionary lineages, geographically separated and displaying high levels of island endemicity. Geographically close populations such as Porto Santo and Madeira (Madeira Archipelago) or Tenerife and Gran Canaria (the Canary Islands) were found to be structured, despite the impossibility of identifying potential physical barriers, especially over a time of millions of years since the establishment of founding populations. Intra-specific density-dependent priority effects have been shown to limit individual dispersal success in populations undergoing range expansion over a range of geographical and temporal scales [85]. Such mechanisms may be particularly relevant for *C. calyculata*, a brooder which, by producing local established populations, would contribute with a higher introgression of local genes when interbreeding with later-arrivals occurs. Although species-specific populations growth and reproductive rates should be investigated to further assess the impact of such mechanisms, density-dependent priority effects have been shown to significantly influence population structure, causing even frequent dispersal events to be unsuccessful ( [84] and references therein).

In our study, the high COI genetic differentiation between the archipelagos and the Mediterranean reaches 8%. DNA barcoding with COI sequences has been proven efficient for the identification of known species and the discovery of overlooked taxa, namely cryptic species in several molluscan families [86]. Nevertheless, various thresholds for species differentiation have been suggested, e.g., 3% divergence [87], or 10x the average intraspecific divergence [88]. Layton et al. [86] found 11 marine molluscan taxa with intraspecific divergence greater than 2% and suggested the possible presence of two different species in the case of *Clione limacina* (Phipps, 1774) in the Arctic Ocean, with a divergence of 5.9%. Thus, the observed degree of divergence might indicate the presence of cryptic species of *C. calyculata* in the NE Atlantic. If haplotype diagrams do not appear as a single network, such could be an indication of the presence of different species [89]. This is not surprising since, for marine taxa, studies on over 33,000 species suggest over 37% remain undescribed [90]. The existence of different species in the three archipelagos, is consistent with the distances between mtDNA haplotypes and the high level of genotypic differentiation between the archipelagos. It could also explain the high level of missing data and null alleles in the dataset.

Bivalves constitute a large and diverse group and imposing a particular delimitation for species differentiation might cause large error rates as has been proven on large data sets with significant overlaps between intra- and interspecific distances [91]. Conclusions on the presence of cryptic species of *C. calyculata* in the NE Atlantic must thus be cautious. Such inferences are further inhibited by the low representation of COI samples from this study (only 12 due to technical limitations), thereby leading the discussion to centre upon the population genetic patterns inferred from the SSR-GBAS data. Nevertheless, the resulting COI patterns additionally shed light on the relevant role of the Canary Islands and the Azores Archipelago in maintaining the genetic heritage of certain marine taxa in the NE Atlantic over geological times [84].

### Oceanic islands as drivers of genetic differentiation

The high degree of divergence found among archipelagos and the mainland as explained by the pairwise FST divergence between archipelagos (Table 3), and the high evolutionary divergence amongst sequences suggesting the potential existence of cryptic species (Table S.5), indicate that lineages of *C. calyculata* occurred in these archipelagos for a long time and were not extirpated during the pronounced changes associated with glacial/interglacial cycles. Macaronesian islands have been suggested to have acted as refugia for several marine organisms during the Quaternary glacial periods (e.g [92–96]. , and together with the Iberian Peninsula and the Mediterranean have been included in a list of potential LGM marine refugia in the NE Atlantic [97]. As pointed out by Ávila et al. [92]. , who combined their palaeontological data with ecological and genetic data from other authors [94, 98, 99], the most probable hypothesis supports the survival of most (if not all) temperate and subtropical species in Azores during the Last Glacial episode, as long as their ecological traits were not constrained to sandy habitats. *Cardita calyculata* lives associated with hard grounds and, as demonstrated by Ávila and co-workers, most species with similar ecological traits were able to cope with the effects of glacial episodes (see [23] and references therein for a resume). The direct effects, e.g. the lowering of SSTs, as well as indirect effects, like the shortening of the time for reproduction and viable development of offspring [100], or the drop of sea level [101–103], had been overcome by the species unscathed.

The oldest fossil record of *C. calyculata* comes from Lower to Middle Miocene assemblages from the Torino Hills, Italy [104]. This species is also reported from the Lower Pliocene of Azores [105] and the Canary Islands (Lanzarote, Fuerteventura and Gran Canaria [106]), ; the Pleistocene of the Canary Islands and Porto Santo (Madeira Archipelago; [25]); and from the Last Interglacial (LIG) fossil record of Azores [107, 108], Selvagens [109], Canary Islands [106] and Cabo Verde [25]. Today, it is reported for all Macaronesian archipelagos [110, 111]. In the oldest Azorean Island, Santa Maria, the two Pliocene outcrops from where this bivalve species is reported (Ávila, unpublished data) are Pedra-que-pica (4.78 ± 0.13 Ma to 4.13 ± 0.19 Ma) and Ponta do Castelo (4.13 ± 0.19 Ma to 3.98 ± 0.05 Ma) [112]. The age of fossil deposits in the Canaries is 5.0 to 4.1 Ma [113], similarly to the records from Santa Maria (Azores). Combined with our genetic data, this suggests that the colonization of the archipelagos might have been more or less contemporaneous with each other. During “windows of opportunity”, e.g., the final period of glacial terminations as first hypothesized by Ávila [114] and later expanded by Ávila et al. [21, 23, 24]. , or during the early stages of the interglacials, as suggested by Ávila et al. [24]. , Muhs et al. [115], and Meco et al. [116], , many marine insular species expanded their geographical ranges towards higher latitudes as demonstrated by the fossil record of the Last Interglacial in both the Canary Islands and the Azores [23, 25, 26, 117–119]. During the last one million years, such “windows of opportunity” have occurred over ten times, providing repeated chances for gene flow between the Macaronesian archipelagos. This phenomenon possibly prevented speciation events, explaining the persistence of species such as *C*. *calyculata* in these insular ecosystems for the last 5 Myr.

Therefore, we do not interpret the significant genetic structure of *C. calyculata* throughout our study system to be consequent to a hypothetical reduction of the species geographical range throughout the NE Atlantic during glacial events, but rather an effect of the geographical isolation of the studied archipelagos that might pose significant restrictions for gene flow across populations. Consequently, random genetic drift creates unique alleles and monophyletic groups in isolated populations with high levels of genetic diversity and high dissimilarity to other populations [97]. Glacial periods and recurrent isolation and lower connectivity between populations lead to stepwise increases of accumulation of differences in allele frequencies, and thus between populations which would be still considered of the same species. Within this scenario, species specific factors such as dispersal ability play an essential role in the demographic responses of a population to range expansions and contractions thus determining current population structures [120]. Population size also affects the speed of the outcome and, considering that low-latitude populations and southern refugia are known to maintain higher viable populations, these would result in higher proportions of unique and localized haplotypes [95]. Similarly, our study shows that even when only a sub-sample of the populations in the Canary Islands are analysed (La Graciosa, Lanzarote and Fuerteventura islands, which were a single island, and were thus connected in the LGM), significant genetic structuring is found as reflected from both the microsatellite pattern and the high evolutionary divergence values within COI sequences.

### Ecological-climatic interactions

Although very rare, extreme geographically restricted endemism cases are known in the marine realm (e.g., *Conus* gastropods in Cabo Verde; [121, 122]). In these cases, local barriers are hypothesised to have a more significant impact by restricting the species’ geographical range, reducing gene flow between populations, and consequently promoting the radiation of the taxa within the archipelago [8, 123, 124]. In nearby archipelagos (e.g., Azores/Madeira, or Selvagens/Canary Islands), species with non-planktotrophic modes of larval development might speciate in one of the archipelagos and later disperse and successfully establish on others (12,21).

The significant population genetic structure inferred from the genotype dataset suggests that *Cardita* individuals were able to disperse throughout the NE Atlantic in the past. However, as populations from different NE Atlantic Archipelagos clearly diverge from each other, long-distance dispersal opportunities might have been limited in number and time, associated with sweepstake routes operating during “windows of opportunity” [125]. *Cardita calyculata* broods its embryos, thereby protecting them during the early stages of development and increasing the survival rate. Altogether, the brooding nature of *C. calyculata* might explain the high level of genetic structuring detected in the studied populations. Additionally, its widespread distribution might be consequent to a high environmental tolerance of this species which, at times when successful dispersal might have been difficult, enable isolated population to survive and diversify into unique haplotypes.

Environmental tolerance plays an important role in determining species survival and geographic ranges [125–128]. Marine species that are able to withstand intertidal conditions, with periodic drastic variations in temperature and humidity, have developed various stress-protection mechanisms for their self-protection and preservation [129]. The greater physiological performances of intertidal organisms reflect their evolutional adaptation to local environments and this might be a pre-adaptation to cope with climate-related temperature changes [130]. Additionally, epibenthic rocky-shore intertidal organisms have been shown to be the best candidates for rafting (e.g., the transport of egg capsules or byssate bivalves attached to floating objects), which provides an important mean of dispersal for species with shorter or non-existing planktotrophic larva stages [1, 11, 21, 131–133]. Especially for marine benthic intertidal species with non-planktotrophic development, rafting has been suggested to be an important dispersal mechanism within oceanic islands [8, 11]. Differently from continents, in which provide dispersal by stepwise adult migration benefits from physical continuity of land mass, oceanic islands are separated by deep waters that constitute effective dispersal barriers for species with no planktotrophic larval stages. Within insular environments species associated with hard substrata or rocky shores covered by algae are optimal candidates for rafting opportunities when compared to deeper infaunal species [1, 13]. Moreover, Ávila et al. [8]. , recognized a direct relationship between bathymetry and geographical range, showing three out of eight prosobranch species from the Azores inhabiting the intertidal environment (and with no planktotrophic dispersal), having the widest geographical ranges. Such dispersal mechanisms may thus have permitted *C. calyculata* to have reached distant archipelagos within Macaronesia during favourable oceanographic conditions and later potentially diverged within a particular archipelago once climatic conditions restricted its latitudinal range and favoured local adaption.

## Conclusions

Despite glacial periods being the main drivers of various faunal extirpations and even extinctions, in the marine realm they were also essential in creating potential stepping-stone routes for marine dispersal. Connectivity between oceanic islands within archipelagos, and between archipelagos and continental landmasses has been significantly altered through geological time, consequently shaping today’s biodiversity patterns [77, 134]. The high genetic differentiation between the NE Atlantic archipelagos and the Mediterranean, as found in this study, indicates relevant roles of the Canary archipelago (and potentially the Azores), acting as a “museum” during glacial times (allowing the long-term persistence of hard ground-associated species) and as a “cradle” (promoting the genetic diversity of marine species, and, in some cases, the formation of *de-novo* species during interglacial times, when larger insular littoral area promotes higher speciation rates [21].

Biodiversity-rich spots in the Northern Hemisphere have been found to be disproportionally more affected than those in the Southern Hemisphere due to the latitudinal pattern of warming [135]. Thus, knowledge retrieved from Northern Hemisphere biogeographic studies such as the present one should drive the design of effective “Natural Protected Areas” of key oceanic habitats to improve the protection of viable genetic diversity. The hereby studied archipelagos represent important habitats connecting and supporting the genetic diversity of *C. calyculata* through geological time in the NE Atlantic. As the overexploitation of marine resources and the ever-increasing human induced climatic shifts has been causing the extirpation of keystone species in trophic chains [136–138], the preservation of such high genetic diversity might be essential for the survival and resilience of marine species and ecosystems.

Finally, we stress that the essential biogeography and evolutionary role of oceanic islands in the NE Atlantic for *C. calyculata* and other marine species, referenced herein, calls for the implementation of a direct conservation management of these environments.

## Electronic supplementary material

Below is the link to the electronic supplementary material.

Supplementary Material 1

## Data Availability

The datasets generated and/or analysed during the current study are available in the Genbank repository, BioProject ID: PRJNA1099938.

## Abbreviations

AMOVA: Analysis of molecular variance
AZ: Azores
bp: base pair
CI: Canary Islands
EDTA: Ethylenediaminetetraacetic acid
GRX: Granada
MCMC: Markov chain Monte Carlo
MD: Madeira
COI: Cytochrome c oxidase subunit 1
DBUA: Department of Biology of the University of the Azores
DNA: Deoxyribonucleic acid
FUE: Fuerteventura
gDNA: genomic DNA
GBAS: Genotyping by amplicon sequencing
HCl: Hydrogen chloride
Ho: Observed heterozygosity
HWE: Hardy-Weinberg equilibrium
LAG: La Graciosa
LAN: Lanzarote
LGM: Last Glacial Maxima
Myr: Million years
mtDNA: mitochondrial DNA
MED: Mediterranean
Mono: Monomorphic
NE: north-east
PCR: Polymerase chain reaction
PIX: Pico
PE: paired-end
SDS: Sodium dodecyl sulphate
SE: Standard error
SMG: São Miguel
SNP: Single nucleotide polymorphism
SSR: Single sequence repeat
SST: Sea surface temperature
SJZ: São Jorge
TBE: Transfer bootstrap expectation
Tris: Tris(hydroxymethyl)aminomethane
uHe: unbiased expected heterozygosity
V: Volt
WAI: Whole amplicon information
