# Supplementary material for: Oceanic islands act as drivers for the genetic diversity of marine species: Cardita calyculata (Linnaeus, 1758) in the NE Atlantic as a case-study
Source: BMC Ecol Evol. 2024 Nov 7;24:138. doi: 10.1186/s12862-024-02322-2 (PMC11542354; doi:10.1186/s12862-024-02322-2)
Supplement: Supplementary file 1 — Supplementary Material 1 [file 12862_2024_2322_MOESM1_ESM.docx]

**Supplementary Material**

**The evolutionary and biogeographic relevance of oceanic islands in the NE Atlantic: a population genetic study of the marine bivalve *Cardita calyculata* (Linnaeus, 1758)**

Sinigaglia L^1,2,3,4^, Baptista L^2,3,7^, Alves C^1,2,3,4^, Feldmann F^1,2,3,4^, Sacchetti C^1,2,3,4^, Rupprecht C^1^, Vijayan T^1^, Martín-González E^8^, Ávila SP^2,3,4,5^, Santos AM^3,6^, Curto M^1,6^, Meimberg H^1^

^1^ Institute for Integrative Nature Conservation Research, University of Natural Resources and Life Sciences (BOKU), Vienna, Austria;

^2^ CIBIO, Centro de Investigação em Biodiversidade e Recursos Genéticos, InBIO Laboratório Associado, Pólo dos Açores, 9501-801 Ponta Delgada, Azores, Portugal;

^3^ MPB-Marine Palaeontology and Biogeography lab, University of the Azores, Rua da Mãe de Deus, 9501-801 Ponta Delgada, Azores, Portugal;

^4^ Faculdade de Ciências da Universidade do Porto, Rua do Campo Alegre 1021/1055, 4169-007 Porto, Portugal;

^5^ Departamento de Biologia, Faculdade de Ciências e Tecnologia, Universidade dos Açores, 9501-801 Ponta Delgada, Azores, Portugal;

^6^ CIBIO, Centro de Investigação em Biodiversidade e Recursos Genéticos, InBIO Laboratório Associado, Universidade do Porto, Campus de Vairão, Rua Padre Armando Quintas, no. 7, 4485-661 Vairão, Portugal;

^7^ NIOZ Royal Netherlands Institute for Sea Research, Landsdiep 4 1797 SZ 't Horntje, Netherlands;

^8^ Museo de Ciencias Naturales de Tenerife, Organismo Autónomo de Museos y Centros, C/ Fuente Morales, 1, 38003 Santa Cruz de Tenerife, Canary Islands, Spain

*DNA extraction protocol (Section 2.2)*

The ethanol-embedded tissue was dried in a paper towel and transferred into a 2 mL tube for the extraction process.

1) 400µLof lysis buffer (2% SDS, 2% PVP 40, 250 Mm NaCl, 200 Mm Tris HCl, 5 Mm EDTA, pH= 8.0) + 13 µL of Proteinase K (10 mg/mL) added to each sample. The solution was shortly vortexed and left in a thermomixer overnight (56 °C, 300 rpm).

2) 13 µL of RNase (10 mg/mL) added to the solution, vortexed and incubated for 15 minutes in the thermomixer (37 °C, 300 rpm). 100 µL of 3 M potassium acetate (pH=4.7), - stored at -20 °C-, added to the solution.

3) Stepwise centrifugation (ThermoScientific, Heraeus Multifuge X3R Centrifuge) as follows: 1000 rpm (1 min), 2000 rpm (1 min), 4000 rpm (1 min), 8000 rpm (1 min), 11000 rpm (7 min).

4) 400 µLof supernatant was transferred to a 96 well, 2 mL deepwell plate containing 600 µL of binding buffer (Composition: 237.5 ml EtOH absolute, 12.5 mL H₂0, 47.756 g guanidine hydrochloride, pH= 5.5). The solution was mixed 10 times and 400 µL were transferred to a 96 well silica membrane EconoSpin® plate.

5) Stepwise centrifugation as follows: 500 rpm (2 min), 4000 rpm (5 min), 6000 rpm (5 min); flow-through solution was discarded.

6) Steps 4 and 5 repeated with the remaining solution.

7) Two ethanol washing-steps were conducted: 600 µL of 80% ethanol added to the EconoSpin® plate and centrifuged at 4000 rpm (2 min), flow-through was discarded. This step repeated with centrifugation at 6000 rpm (2 min).

8) Ethanol traces were removed by drying the EconoSpin® plate for 15 min at room temperature to ensure its complete evaporation.

9) Elution on a 96-well plate: first elution step by adding 30 µL of elution buffer (10 Nm Tris, pH= 8.0) and centrifuging at 6000 rpm (3 min). Second elution step in a new 96-well plate by adding 50 µL of Elution buffer (10 Nm Tris, pH= 8.0) and centrifuging at 6000 rpm (3 min). Both flow-through solutions containing DNA were preserved at -20 °C.

*Laboratorial procedures for Illumina sequencing (Section 2.3.1 and 2.3.2)*

Recognition sequences were designed to be added to both forward (P5 - TCTTTCCCTACACGACGCTCTTCCGATCT) and reverse primers (P7 - CTGGAGTTCAGACGTGTGCTCTTCCGATCT) to serve as linkers during a second Index-PCR using primers containing the eight bp indexes and TrueSeq adapters (P5: AATGATACGGCGACCACCGAGATCTACAC [Index] ACACTCTTTCCCTACACGACG; and P7: CAAGCAGAAGACGGCATACGAGAT [Index] TGACTGGAGTTCAGACGTGT). Adapters are ligated though A-tailing and unique indexes per sample, as well as flow-cell binding motifs, are added with a PCR. These indexes are used by the Illumina machine to demultiplex when the fastq files are created. Fragments are sequenced in four steps: Read1, Index 1, Index 2, Read 2.

PCR clean-up was conducted with the Agencourt AMPure XP PCR Purification kit (Beckman Coulter Inc., Bree, CA, USA), as follows. A volume of 4.3 µL of AMPure XP beads was mixed with the 6 µL of pooled PCR product and incubated for 5 min at room temperature. The DNA binds to the magnetic beads after the first step, which were then captured by an inverted magnetic bead extraction device (VP407‐AM‐N, by V&P Scientific, INC.) and washed two times for 45 s in 200 µL of 80% ethanol. After drying the beads at room temperature for 5 min, the DNA was eluted in 17 µL of elution buffer (10 mM Tris–HCl, pH 8.3) at 65 °C.

*SSR-GBAS marker development (Section 2.3.1)*

The sequence reads, after quality check with FastQC v0.11.9 (55), were processed with Trimmomatic v0.39 (56) to trim adapters and low-quality regions (Phred score > 20) in forward and reverse reads, which were then merged using Usearch v11 (57). The SSR_pipeline’s script SSR_search.py (58) was used to preferentially isolate sequences containing 5- and 4-mers repeats but some 3-mers repeats were also accepted. A minimum of 30 bp flanking regions on both sides of the motif and a medium of 6 repetitions for 3mers, 5 repetitions for 4mers and 4 repetitions for 5mers were set as parameters during the SSR search. The sequences identified as containing desired SSR-GBAS motifs were manually checked to remove those containing more than one motif, interrupted motifs and long mononuclear stretches (>6 bp). Primers were designed using Primer3 as implemented in Geneious v2022.2.2 (59) using the default parameters and targeting a product size between 400 and 500 bp. Moreover, to avoid problems in the overlap of the paired reads, only primers producing amplicons containing the repetition motifs either in the first or last 300 bp were selected (53). Primers were extended with a part of the Illumina adapter as described in Curto *et al.* (53) for amplicon library preparation via a second PCR run.

The primers designed from the initial Illumina MiSeq run were individually tested using gDNA of two specimens of *C. calyculata*, in PCR reactions conducted for a final volume of 10 µl: 5 µL of QIAGEN Multiplex PCR Master Mix (Qiagen, CA, USA), 1 µL of each primer at 1 μM, 1 μL of gDNA and 3 µL of water. The cycling conditions were as follows: 95 °C for 15 min; 30 cycles of 95 °C for 30 s, 55 °C for 1 min, and 72 °C for 1 min; final extension at 72 °C for 10 min. An electrophoretic run in 1.5% agarose gel at 80 V allowed the visualization of the amplicons.

*Molecular marker amplification –Sequencing of mtDNA (COI) (Section 2.4.1)*

For 20 µL reactions: 10 µL of QIAGEN Multiplex PCR Master Mix (Qiagen, CA, USA), 2 µL of gDNA, 4 µL of each primer (2 μM). For the 10 µL reactions all reagents but gDNA (3 µL) were scaled down to half.

The following cycling profile was applied to both reaction volumes: 95 °C for 15 min; 35 cycles of 95 °C for 30 s, 50 °C for 1 min, and 72 °C for 1 min; 72 °C for 10 min. Amplification was checked in an electrophoretic run in 2% agarose gel at 250 V. The 20 µL were sent for Sanger sequencing at AGENTA Genewiz (Leipzig, Germany) producing reads in both directions. The 10 µL were sequenced at the Centre for Molecular Analyses (CTM from CIBIO-InBIO Research Centre, Vairão, Portugal). To this end, 8 µL of PCR product was cleaned with 1µL ExoSap Product Clean-up Reagent (Exonuclease from New England BioLabs Inc.: 20000 U/mL + SAP from Affymetrix: 1uM/u) through an incubation 37 °C for 15 min followed by an enzymatic inactivation at 80° C for 15 min. The final PCR product was diluted 1:1. The following temperature profile was used: 95 °C for 30 min; 24 cycles of 96 °C for 10 s, 50 °C for 5 s, 60 °C for 4 min. Sequencing was conducted at the Centre for Molecular Analyses (CTM from CIBIO-InBIO Research Centre, Vairão, Portugal).

*Parameters for mtDNA COI analysis (Section 2.4.2)*

Multiple-alignment of the COI dataset with Geneious Prime v2022.2.2 with following parameters: Consensus Alignment, Alignment Type: Global alignment with free end gaps, Cost Matrix: 65% similarity, 5.0/-4.0.

Raxml-ng code: Raxml-ng --all --msa *Cardita*_var_ita.phy --model partitions.txt --prefix R5 --threads 2 --seed 2 --bs-metric fbp,tbe --outgroup GQ166578.1; Partitions.txt: TVM+I, gene1codon1andcodon2 = 1-654\3, 2-654\3; TVM+I, gene1codon3 = 3-654\3.

**Table S1** *-* **Complete list of all SSR-GBAS primers designed in this study**. Includes sequence of forward and reverse primers (5’-3’), the repetition motif, and number of repeats in the original sequence used for the primer design, the primer-mix in which it was included for the Multiplex PCR, and Information regarding failed primers.

| **Primer/Loci** | **Repetition motif** | **Forward (5’-3’)** | **Reverse (5’-3’)** | **Mix** | **Info** |
| --- | --- | --- | --- | --- | --- |
| 1_CC18 | 8(TATT) | CCTGGGTCATAACAAACTTA | GATAGTGCCCCAATGGTAAT | 1 | Failed (>50 % missing data) |
| 2_CC18 | 6(AATC) | AACGCTGTGTTGTCTTCT | AATGTTCACAGTGAAGATATGA | 1 |  |
| 3_CC18 | 4(AAAC) | ATTGCTGAACTCGACCCATA | CAATGGATTCCAACCTTCAA |  | Failed in single PCR test |
| 4_CC18 | 4(AGTG) | GTCTGCAGATCTGTTCATTG | AGGTGGAGAAAAAGCAATGA |  | Failed in single PCR test |
| 5_CC18 | 8(CTTA) | AACGTAACGTCACGACATAA | AGTTTGAATTTATTTACGCGTATA | 1 |  |
| 6_CC18 | 4(GTCT) | CTTTCGCAGCCAAATAACAA | ATGGTGAACTTTGCCCATAC | 1 | Failed (>50 % missing data) |
| 7_CC18 | 5(GACA) | CCATTGGGTGTAATGTTGAA | AATGAGTGGATGAGCACAAT | 1 | Failed (>50 % missing data) |
| 8_CC18 | 4(TGAG) | ACAAAATTCACTGGCTCAAT | ACTATCTGTCCACTGCAGTA |  | Failed in single PCR test |
| 9_CC18 | 5(TATT) | CGTTGGTACGTCGTTTTTAG | GCCATCAATCCCATCTCTTC | 1 | Failed (>50 % missing data) |
| 10_CC18 | 4(CGGA) | GTGGAGGCATAAGGATTGAT | TTTCGGTGGAATAGCTTAAA | 1 |  |
| 11_CC18 | 4(TCAAC) | CTGCGAGGTAAACTGTGTAC | GACTACACCTCACCTCATAAC | 1 |  |
| 12_CC18 | 4(ATTAA) | AGGTGGTAGAAAAGCAATTC | GATATTCCTTCGTACAGCAATG | 1 |  |
| 13_CC18 | 4(AGTTC) | CCCTCGTTAACCCCCTAATA | TGAATAACTCTGTTCTGAAAATT | 1 |  |
| 14_CC18 | 4(AATCG) | GCCCAACCATTGTTCATAGT | CCAAAGGCAGAGTGTCATAT | 1 | Failed (>50 % missing data) |
| 15_CC18 | 4(GGAAA) | GCGACCCATTACTTACTCTT | ACTGACTGACTGACTGACTA |  | Failed in single PCR test |
| 16_CC18 | 5(TTTTG) | GCTGTCGTGATTGTGAAATT | CAAACCTGCTAAATCGAAACA | 1 | Failed (>50 % missing data) |
| 17_CC18 | 4(ACAAA) | TGATACTAGCACCAAGTTCAT | ACCCTAACCAACTGGAGATA |  | Failed (>50 % missing data) |
| 18_CC18 | 4(GAACA) | TACGTTGTCACAGCCTTAAA | GAACCGTTGTTCTTAAAATGT | 2 |  |
| 19_CC18 | 4(AAACA) | GGGGTGCAGTTAATGAATTAC | GCCATTGATGTGTGTTTCTT | 2 |  |
| 20_CC18 | 4(ACTGC) | CCTAAAGGTGCTGGTAAGAG | GCAGAGCCTAATCCATAGAG | 2 | Failed (>50 % missing data) |
| 21_CC18 | 5(GAATT) | CAAGGTCATGAGGTCTGATC | TTACCCTACGTAAAATGCTAT | 2 |  |
| 22_CC18 | 4(TTTTA) | CGATTTCCGCGCTTATTAAG | TGTGGTAAGACGACAATTTG | 2 |  |
| 23_CC18 | 6(ACAGA) | CCAAAGTTGGTAGACGTTTT | ACAATACTTGTATACGATGATTTG | 2 |  |
| 24_CC18 | 4(GGGGT) | TCAAGGCAGTGCTACAATAA | ACACATTCAACCAAAATTAGGA | 2 | Failed (>50 % missing data) |
| 25_CC18 | 6(GGCTC) | CACCATCACCTCTGGTATTG | TAGCCCCAATCCATAGTGTA | 2 | Failed (>50 % missing data) |
| 26_CC18 | 5(TTCG) | AGGCATGTTCTTTTGCATTA | ATATACTCAACTGCCTGAACAA | 2 |  |
| 27_CC18 | 5(AAAT) | ATATACTCAACTGCCTGAACAA | CAAACAAGTGTTGCCAAAGA |  | Failed in single PCR test |
| 28_CC18 | 4(TAAA) | AACACTTTGGTTTGCACATA | CCTCCACATCCCTAAAAGAT | 2 | Failed (>50 % missing data) |
| 29_CC18 | 5(AAA) | CCTCCACATCCCTAAAAGAT | TTCGCTTCTTCGTGATCATA |  | Failed in single PCR test |
| 30_CC18 | 4(TTTG) | AGAGGATGTTGCAGGAAAAT | CAATCCGTGCTAAACCTTTC | 2 | Failed (>50 % missing data) |
| 31_CC18 | 7(TATG) | CAATCCGTGCTAAACCTTTC | TGAGTTGTAATGATATACATGAAA | 2 |  |
| 32_CC18 | 5(GTCC) | AGTTGTCTATGAGATACTAGTATT | CAACACGAGTCCTCCTAAAA | 3 | Failed (>50 % missing data) |
| 33_CC18 | 4(ACAA) | CAACACGAGTCCTCCTAAAA | CGTTTAATTTCCCATTGCCATA | 3 | Failed (>50 % missing data) |
| 34_CC18 | 6(CCAA) | TTTTCCCATCCCTCCTTCTT | CGCCTCGAATTTAACATCTT |  | Failed in single PCR test |
| 35_CC18 | 6(ACAG) | CGCCTCGAATTTAACATCTT | ACATTTCACTTACCACTTACA |  | Failed in single PCR test |
| 36_CC18 | 5(CCCG) | ACTGAAACATGGAAGCAATC | AGCTCATCTCCCTAGTTGTA | 3 |  |
| 37_CC18 | 4(ACTC) | AGCTCATCTCCCTAGTTGTA | CGAATTGCTGAATCCGTTAG |  | Failed in single PCR test |
| 38_CC18 | 5(ATAG) | ACGAATCCTGGTAAGGTATG | ACAGGACTGTTACATTACTATTC |  | Failed in single PCR test |
| 39_CC18 | 5(ATTT) | ACAGGACTGTTACATTACTATTC | GCCGATTGATACGTTGATTT | 3 | Failed (>50 % missing data) |
| 40_CC18 | 7(GATA) | TCCCACTGAAAGCAAAGAAG | CTCCACGGACAAATTCAAAT |  | Failed in single PCR test |
| 41_CC18 | 4(TTAT) | GCTCGTGACGTTTTACTTTT | ACACCAGCGAAATACTAAGT |  | Failed in single PCR test |
| 42_CC18 | 4(TTATT) | ACTTTTGTAGTCTTCAGCATTA | GCCAAAGCTCAAGAAAAACA | 3 |  |
| 43_CC18 | 4(GTGTT) | AGTTCAGTGATTGTGTTGAT | ATCGGCAAAGAAGGAAAAAG | 3 |  |
| 44_CC18 | 4(ACCTG) | TTTACCAAGCCACCATTTTT | ACCACTAAATTTCCTGTATCTT |  | Failed in single PCR test |
| 45_CC18 | 4(TTTGC) | ATGGTGGCATTTGCAGTAC | TGTTACTTTTCGTGCGATTC | 3 |  |
| 46_CC18 | 4(ATTCA) | ACTCCGTCTATAGTGATCCTA | TACAGTTCACAAAGCTCAAA |  | Failed in single PCR test |
| 47_CC18 | 4(CTCCC) | GGCTTGTGTCGCGATATAA | CAAGGTGGCATTTCATGAAA | 3 | Failed (>50 % missing data) |
| 48_CC18 | 4(GGGTG) | ACTCAATTTAAAGGGAAGCACTA | TATTTTTGATGAAAGTATGCCATA | 3 |  |
| 49_CC18 | 8(TTGTA) | TGTGTGTCAATTTGCAATGT | GCAGGCAAAAGAGAAGAAAA | 3 | Failed (>50 % missing data) |
| 50_CC18 | T4(TTTC) | TAAGGATCCAAGCACACAAT | TTCACGGTGCGTCACTTA | 3 | Failed (>50 % missing data) |

**Table S2.** Mitochondrial DNA (mtDNA) cytochrome *c*-oxidase subunit 1 (COI) sequences of *Cardita variegata* (Bruguière, 1792) and *C. calyculata* (Linnaeus, 1758) retrieved from GenBank and from this study (*). AZ – Azores; CI – Canary Islands; MED – Mediterranean.

| **Species** | **Ascension Number** | **Origin** |
| --- | --- | --- |
| Cardita variegata | GQ166578 |  |
| Cardita calyculata | AF120660 | Mediterranean, Spain |
| Cardita calyculata | KP068100 | Roses, Girona, Spain |
| Cardita calyculata | KC429112 | Mediterranean, Spain |
| Cardita calyculata | MT920124 | Javorika, Veliki Brijun, Croatia |
| Cardita calyculata | MT920125 | Javorika, Veliki Brijun, Croatia |
| Cardita calyculata | MT920126 | Javorika, Veliki Brijun, Croatia |
| Cardita calyculata | MT920127 | Javorika, Veliki Brijun, Croatia |
| Cardita calyculata | MT920128 | Javorika, Veliki Brijun, Croatia |
| Cardita calyculata | MT920129 | Javorika, Veliki Brijun, Croatia |
| Cardita calyculata | CC7 | Lajes do Pico, Pico, AZO |
| Cardita calyculata | CC9 | Lajes do Pico, Pico, AZO |
| Cardita calyculata | CC239 | Playa del Hierro, Fuerteventura, CAN |
| Cardita calyculata | CC361 | Baja de Bristol, Fuerteventura, CAN |
| Cardita calyculata | CC370 | Baja de Bristol, Fuerteventura, CAN |
| Cardita calyculata | CC374 | Baja de Bristol, Fuerteventura, CAN |
| Cardita calyculata | CC384 | Playa de la Barra, Fuerteventura, CAN |
| Cardita calyculata | CC390 | Playa de la Cocina, La Graciosa, CAN |
| Cardita calyculata | CC391 | Playa de la Cocina, La Graciosa, CAN |
| Cardita calyculata | CC393 | Playa de la Cocina, La Graciosa, CAN |
| Cardita calyculata | CC394 | Playa de la Cocina, La Graciosa, CAN |
| Cardita calyculata | CC447 | Playa Quemada, Lanzarote, CAN |

**Table S3.** Estimates of Hardy–Weinberg Equilibrium (HWE) and Wright’s Fixation index (F). Analysis per marker and population with at least five individuals. Significant (* p < 0.05, ** p < 0.01, *** p< 0.001) deviations to HWE are highlighted in bold. Monomorphic loci (Mono) are also reported. GRX: Granada, Spain. AZO: Azores Archipelago. MAD: Madeira Archipelago. CAN: Canaries Archipelago. LAG – La Graciosa Island. LAN: Lanzarote Island. FUE: Fuerteventura Island. PIX: Pico Island. SJZ: São Jorge Island.

| **Locus** | **CàletonGRX, SPAIN** |  | **Playa de la Cocina, LAG - CAN** |  | **Pechiguera, LAN - CAN** |  | **Playa Quemada, LAN - CAN** |  | **Baja de Bristol, FUE - CAN** |  | **Los Lapios, FUE - CAN** |  | **Playa del Hierro, FUE - CAN** |  | **Playa de la Barra, FUE - CAN** |  | **Salinas Câmara de Lobos, MAD - MAD** |  | **Lajes do Pico, PIX - AZO** |  | **Velas, SJZ - AZO** |  |
| --- | --- | --- | --- | --- | --- | --- | --- | --- | --- | --- | --- | --- | --- | --- | --- | --- | --- | --- | --- | --- | --- | --- |
|  | **HWE** | **F** | **HWE** | **F** | **HWE** | **F** | **HWE** | **F** | **HWE** | **F** | **HWE** | **F** | **HWE** | **F** | **HWE** | **F** | **HWE** | **F** | **HWE** | **F** | **HWE** | **F** |
| **11** | 0.896 | -0.266 | **0.012** | 0.717 | **0.027** | 0.425 | **0** | 0.348 | 0.096 | 0.341 | **0.033** | 0.216 | **0** | 0.694 | **0.02** | 0.391 | 0.157 | -1 | **0.013** | 0.046 | 0.82 | 0.052 |
| **13** | **0.003** | 0.608 | 0.188 | 0.256 | 0.95 | 0.072 | 0.926 | -0.132 | 0.849 | -0.273 | 0.998 | -0.812 | 0.503 | -0.119 | 0.736 | 0.067 | Mono |  | 0.919 | -0.02 | 1 | -0.067 |
| **43** | **0.02** | 0.137 | **0.005** | 0.814 | **0** | 0.429 | **0** | 0.846 | 0.255 | 0.208 | **0** | 0.709 | **0** | 0.588 | **0.029** | 0.736 | Mono |  | **0** | 0.354 | **0** | 0.579 |
| **12** | 0.073 | 0.630 | **0.01** | 0.175 | **0** | -0.035 | **0** | 0.094 | 0.148 | 0.253 | **0.004** | -0.082 | **0.015** | -0.133 | 0.227 | 0.012 | 0.157 | -1 | **0** | -0.204 | **0** | -0.343 |
| **5** | **0.001** | 0.519 | **0.021** | 0.679 | 0.148 | 0.325 | **0** | 0.735 | **0.02** | 0.455 | **0** | 0.554 | 0.178 | 0.289 | 0.113 | 0.744 | 0.572 | -0.6 | 0.967 | -0.059 | 0.999 | -0.026 |
| **10** | 0.995 | -0.148 | 0.132 | 0.395 | **0** | 0.766 | **0.031** | 0.502 | **0.008** | 0.774 | 0.095 | 0.444 | **0** | 0.528 | 0.1 | 0.68 | Mono |  | 0.064 | 0.261 | **0** | 0.4 |
| **22** | 0.414 | 0.333 | **0.038** | 0.234 | **0** | 0.459 | **0.002** | 0.356 | 0.914 | -0.2 | 0.992 | -0.067 | 0.929 | 0.119 | 0.483 | 0.349 | 0.637 | -0.333 | 0.865 | 0.128 | 0.235 | 0.13 |
| **36** | **0.002** | 0.442 | **0.002** | 0.438 | **0** | 0.352 | **0.025** | 0.112 | 0.172 | 0.211 | 0.220 | -0.099 | 0.122 | -0.018 | 0.628 | -0.19 | 0.572 | -0.6 | 0.969 | 0.08 | **0** | 0.363 |
| **23** | **0** | 0.897 | **0** | 1 | **0** | 0.73 | **0.029** | 0.446 | 0.083 | 1 | **0.032** | 0.619 | 0.906 | -0.027 | **0.014** | 1 | 0.637 | -0.333 | **0** | 0.398 | **0** | 0.661 |
| **48** | Mono |  | **0.028** | -0.231 | **0** | 0.561 | 0.921 | -0.025 | 0.71 | -0.429 | 0.926 | 0.12 | 0.981 | -0.061 | 0.573 | -0.4 | 0.637 | -0.333 | 0.102 | 0.284 | **0.013** | 0.309 |
| **42** | **0.001** | 0,862 | **0.011** | 0.368 | **0.007** | 0.609 | **0** | 0.658 | **0.05** | 0.756 | **0.002** | 0.611 | **0** | 0.677 | **0.046** | 1 | 0.637 | -0.333 | 0.458 | 0.258 | **0.017** | 0.311 |
| **19** | 0.955 | -0252 | **0.008** | 0.44 | **0** | 0.09 | **0.002** | 0.293 | **0.05** | 0.268 | **0** | 0.281 | **0** | 0.059 | **0.022** | -0.017 | 0.637 | -0.333 | **0** | -0.107 | **0** | -0.3 |
| **18** | Mono |  | **0.041** | 0.114 | **0** | 0.471 | **0.004** | 0.207 | 0.143 | 0.016 | 0.17 | 0.302 | **0** | 0.19 | **0.003** | 1 | 0.637 | -0.333 | **0** | -0.276 | **0** | -0.21 |
| **21** | 0.296 | -0.226 | Mono |  | 0.222 | 0.2 | **0.018** | 0.565 | Mono |  | **0** | 0.459 | 0.106 | 0.692 | 0.311 | 0.304 | 0.572 | -0.6 | 0.824 | -0.166 | **0.026** | 0.224 |
| **31** | **0.046** | 1 | 0.135 | 0.63 | **0.003** | 0.529 | **0.023** | 0.552 | 0.107 | 0.677 | **0.033** | 0.529 | **0** | 0.89 | 0.081 | 0.442 | Mono |  | 0.973 | -0.014 | **0** | 0.39 |
| **45** | Mono |  | 1 | 0 | 0.348 | 0.163 | 0.794 | -0.014 | 0.06 | 0.2 | 0.126 | 0.054 | 0.691 | 0.07 | 0.677 | -0.143 | Mono |  | Mono |  | **0** | 1 |
| **26** | 0.075 | 0.121 | **0.007** | 1 | **0.004** | 0.594 | 0.054 | 0.292 | 0.112 | 1 | 0.472 | -0.111 | 0.092 | 0.524 | **0.05** | 0.474 | 0.157 | 1 | **0.045** | 0.172 | 0.298 | 0.179 |
| **2** | Mono |  | 0.387 | 0 | **0.083** | 0.623 | **0.002** | 0.145 | 0.172 | 0.385 | 0.144 | 0.234 | 0.127 | 0.261 | 0.157 | 1 | Mono |  | 0.062 | 1 | 0.083 | 1 |
| **16** | **0** | 1 | 0.112 | 1 | 1 | 1 | Mono |  | Mono |  | Mono |  | 0.083 | 1 | 0.157 | 1 | 0.637 | -0.333 | 0.146 | 0.241 | 0.631 | 0.163 |
| **14** | 0.738 | -0.230 | Mono |  | Mono |  | Mono |  | Mono |  | Mono |  | Mono |  | Mono |  | 0.157 | -1 | 0.309 | 0.164 | **0** | 0.701 |
| **25** | Mono |  | **0.046** | 1 | **0.014** | 1 | 0.157 | 1 | Mono |  | **0.046** | 1 | **0.001** | **1** | Mono |  | Mono |  | **0** | 0.879 | **0.007** | 0.453 |

**Table S.4.** Total number of alleles per locus and frequency of null alleles analysed for all populations with more than 5 individuals. Frequency values higher than 0.2 are reported in bold as set as such limit was set for null alleles detection. For other abbreviations, see Table 2.

| Locus | **Total number of alleles** | **Bajas de Bristol, FUE** | **Los Lapios, FUE** | **Playa del Hierro, FUE** | **Playa de la Barra, FUE** | **Playa de la Cocina, LAG** | **Playa Quemada, LAN** | **Pechiguera, LAN** | **Lajes do Pico, PIX** | **Velas, SJZ** | **Caletón, GRX** |
| --- | --- | --- | --- | --- | --- | --- | --- | --- | --- | --- | --- |
| 11-CC18_TCAAC | 34 | 0.15904 | 0.06544 | **0.30947** | 0.16666 | **0.32032** | 0.16735 | 0.19284 | 0.04022 | 0.03571 | 0 |
| 13-CC18_AGTTC | 25 | 0 | 0 | 0 | 0.01168 | 0.09566 | 0.00001 | 0.00836 | 0.00005 | 0.00001 | **0.19702** |
| 43-CC18_GTGTT | 42 | 0.10733 | **0.32883** | **0.23486** | **0.27564** | **0.30522** | **0.35588** | 0.17295 | 0.10340 | **0.22316** | 0.03449 |
| 12-CC18_ATTAA | 45 | 0.09524 | 0 | 0.01755 | 0.00038 | 0.00001 | 0 | 0 | 0 | 0 | **0.21170** |
| 5-CC18_CTTA | 40 | **0.20453** | **0.20527** | 0.13736 | **0.33010** | **0.28047** | **0.33538** | 0.13421 | 0 | 0 | **0.23366** |
| 10-CC18_CGGA | 44 | **0.32589** | 0.19405 | **0.24872** | 0.3 | 0.18316 | 0.19735 | **0.34093** | 0.11329 | 0.17605 | 0 |
| 22-CC18_TTTTA | 25 | 0 | 0.00001 | 0.02239 | 0.11940 | 0.00001 | 0.13713 | 0.12105 | 0.04833 | 0.03224 | 0.11111 |
| 36-CC18_CCCG | 71 | 0.00063 | 0 | 0.01291 | 0 | 0.18853 | 0.04074 | 0.12883 | 0.02738 | **0.15552** | **0.16963** |
| 23-CC18_ACAGA | 26 | **0.31500** | 0.15027 | 0.00004 | **0.25122** | **0.38708** | 0.13413 | 0.14850 | 0.17042 | **0.26212** | **0.40150** |
| 48-CC18_GGGTG | 29 | 0 | 0.01295 | 0.00001 | 0 | 0 | 0 | **0.20943** | 0.12246 | 0.14156 | - |
| 42-CC18_TTATT | 43 | **0.34286** | **0.23597** | **0.31547** | **0.38687** | 0.12660 | **0.21869** | **0.27040** | 0.08952 | 0.13918 | **0.34131** |
| 19-CC18_AAACA | 70 | 0.13333 | 0.00071 | 0.04167 | 0.00041 | 0.16588 | 0.12198 | 0.05559 | 0 | 0 | 0 |
| 18-CC18_GAACA | 48 | 0.00041 | 0.13333 | 0.09423 | **0.32755** | 0 | 0.10052 | **0.20181** | 0.04752 | 0 | - |
| 21-CC18_GAATT | 17 | 0.00100 | 0.00095 | **0.24535** | 0.13173 | 0.00100 | **0.20319** | 0.09391 | 0 | 0.08317 | 0 |
| 31-CC18_TATG | 28 | **0.26881** | **0.20598** | **0.38559** | 0.16526 | **0.20513** | **0.23476** | **0.23191** | 0 | 0.18298 | **0.29029** |
| 45-CC18_TTTGC | 21 | 0.00001 | 0.00001 | 0.06209 | 0 | 0.00058 | 0 | 0.07207 | 0.00100 | 0.19545 | 0.00100 |
| 26-CC18_TTCG | 75 | **0.40000** | 0 | **0.24000** | 0.18900 | **0.34565** | 0.12689 | **0.28271** | 0.08571 | 0.09321 | 0.05269 |
| 2-CC18_AATC | 34 | 0.16667 | 0.12370 | 0.13063 | **0.33333** | 0 | 0.07529 | **0.28097** | **0.42857** | **0.31500** | - |
| 16-CC18_TTTTG | 27 | 0.00100 | - | **0.31500** | **0.33333** | **0.40000** | 0.00100 | **0.31500** | 0.10273 | 0.08655 | **0.43958** |
| 14-CC18_AATCG | 25 | - | - | - | - | - | - | - | 0.07574 | 0.32619 | 0 |
| 25-CC18_GGCTC | 22 | 0.00100 | **0.29029** | **0.41879** | 0.00100 | **0.38687** | **0.33333** | **0.25122** | **0.33792** | 0.19517 | - |

**Table S.5.** Estimates of evolutionary divergence (raw p-distances) between COI sequences of several *Cardita* spp. sequences. The analysis, performed on MEGA v11, considered 20 sequences and 406 bp after excluding positions containing gaps and/or missing data for each sequence pair analysed. Sequences retrieved from GenBank (KP068102 to MT920127), accession Number (AN) and species identification are provided in the table. *Cardita calyculata* COI sequences from this study (CC7, CC9, CC362, CC239, CC393, CC447) were compared to other COI sequences of *C. calyculata* from GenBank (AF120660.1, KP068098.1, KP068099.1, KP068102.1, KP068103.1, MT920125.1, MT920127.1, MT920128.1, MT920129.1) and of *Cardita leana* (KP068102.1, KP068103.1) *Cardita variegata* (MN608301.1, MN608302.1, GQ166578.1), *Cardita thaanumi* (KP068104.1), *Cardita caliculaeformis* (KP068098.1, KP068099.1).

|  | **KP068103.1** | **KP068102.1** | **MN608302.1** | **MN608301.1** | **GQ166578.1** | **KP068104.1** | **KP068098.1** | **KP068099.1** | **AF120660.1** | **MT920125.1** | **MT920128.1** | **MT920129.1** | **MT920127.1** | **CC7** | **CC9** | **CC361** | **CC239** | **CC447** |
| --- | --- | --- | --- | --- | --- | --- | --- | --- | --- | --- | --- | --- | --- | --- | --- | --- | --- | --- |
| **KP068102.1** | 0,00246 |  |  |  |  |  |  |  |  |  |  |  |  |  |  |  |  |  |
| **MN608302.1** | 0,14286 | 0,14039 |  |  |  |  |  |  |  |  |  |  |  |  |  |  |  |  |
| **MN608301.1** | 0,14039 | 0,13793 | 0,02463 |  |  |  |  |  |  |  |  |  |  |  |  |  |  |  |
| **GQ166578.1** | 0,12808 | 0,12562 | 0,03448 | 0,02217 |  |  |  |  |  |  |  |  |  |  |  |  |  |  |
| **KP068104.1** | 0,18719 | 0,18473 | 0,15271 | 0,16502 | 0,15517 |  |  |  |  |  |  |  |  |  |  |  |  |  |
| **KP068098.1** | 0,15517 | 0,15271 | 0,14778 | 0,14532 | 0,15271 | 0,13793 |  |  |  |  |  |  |  |  |  |  |  |  |
| **KP068099.1** | 0,15347 | 0,15099 | 0,14851 | 0,14604 | 0,15347 | 0,14109 | 0,00990 |  |  |  |  |  |  |  |  |  |  |  |
| **AF120660.1** | 0,13793 | 0,13547 | 0,15025 | 0,15271 | 0,13793 | 0,13300 | 0,11823 | 0,12624 |  |  |  |  |  |  |  |  |  |  |
| **MT920125.1** | 0,16502 | 0,16256 | 0,15025 | 0,15271 | 0,14286 | 0,14039 | 0,11823 | 0,12624 | 0,05172 |  |  |  |  |  |  |  |  |  |
| **MT920128.1** | 0,16256 | 0,16010 | 0,14778 | 0,15025 | 0,14039 | 0,14039 | 0,11823 | 0,12624 | 0,05172 | 0,00246 |  |  |  |  |  |  |  |  |
| **MT920129.1** | 0,16502 | 0,16256 | 0,15271 | 0,15517 | 0,14532 | 0,14286 | 0,12069 | 0,12871 | 0,05419 | 0,01478 | 0,01232 |  |  |  |  |  |  |  |
| **MT920127.1** | 0,16256 | 0,16010 | 0,15025 | 0,15271 | 0,14286 | 0,14039 | 0,11823 | 0,12624 | 0,05172 | 0,01232 | 0,00985 | 0,00246 |  |  |  |  |  |  |
| **CC7** | 0,15556 | 0,15309 | 0,16543 | 0,16790 | 0,15309 | 0,15309 | 0,11852 | 0,12655 | 0,05926 | 0,08148 | 0,08148 | 0,08889 | 0,08642 |  |  |  |  |  |
| **CC9** | 0,15062 | 0,14815 | 0,16049 | 0,16296 | 0,14815 | 0,15309 | 0,11852 | 0,12655 | 0,05926 | 0,07654 | 0,07654 | 0,08395 | 0,08148 | 0,00494 |  |  |  |  |
| **CC361** | 0,15271 | 0,15025 | 0,15271 | 0,15517 | 0,14532 | 0,14286 | 0,13054 | 0,13861 | 0,06404 | 0,06158 | 0,06158 | 0,06897 | 0,06650 | 0,05926 | 0,05432 |  |  |  |
| **CC239** | 0,15309 | 0,15062 | 0,15309 | 0,15556 | 0,14568 | 0,14321 | 0,13086 | 0,13896 | 0,06420 | 0,06173 | 0,06173 | 0,06914 | 0,06667 | 0,05693 | 0,05198 | 0,00000 |  |  |
| **CC447** | 0,15271 | 0,15025 | 0,15517 | 0,15764 | 0,14286 | 0,13300 | 0,13054 | 0,13861 | 0,06158 | 0,06404 | 0,06650 | 0,07389 | 0,07143 | 0,05679 | 0,05679 | 0,04680 | 0,04691 |  |
| **CC393** | 0,15517 | 0,15271 | 0,15517 | 0,15764 | 0,14286 | 0,13547 | 0,13054 | 0,13861 | 0,06404 | 0,06650 | 0,06897 | 0,07635 | 0,07389 | 0,05432 | 0,05432 | 0,04187 | 0,04198 | 0,00739 |
